# Haplotypes of single cancer driver genes and their local ancestry in a highly admixed long-lived population of Northeast Brazil

**DOI:** 10.1590/1678-4685-GMB-2021-0172

**Published:** 2022-02-02

**Authors:** Steffany Larissa Galdino Galisa, Priscila Lima Jacob, Allysson Allan de Farias, Renan Barbosa Lemes, Leandro Ucela Alves, Júlia Cristina Leite Nóbrega, Mayana Zatz, Silvana Santos, Mathias Weller

**Affiliations:** 1 Universidade Estadual da Paraíba (UEPB), Núcleo de Estudos em Genética e Educação, Programa de Pós-Graduação em Saúde Pública, Campina Grande, PB, Brazil.; 2 Universidade Estadual da Paraíba (UEPB), Departamento de Biologia, Campina Grande, PB, Brazil.; 3 Universidade de São Paulo (USP), Departamento de Genética e Biologia Evolutiva, São Paulo, SP, Brazil.

**Keywords:** Ancestry, admixed population, cancer driver gene, single nucleotide polymorphism (SNP), haplotype.

## Abstract

Admixed populations have not been examined in detail in cancer genetic studies. Here, we inferred the local ancestry of cancer-associated single nucleotide polymorphisms (SNPs) and haplotypes of a highly admixed Brazilian population. SNP array was used to genotype 73 unrelated individuals aged 80-102 years. Local ancestry inference was performed by merging genotyped regions with phase three data from the 1000 Genomes Project Consortium using RFmix. The average ancestry tract length was 9.12-81.71 megabases. Strong linkage disequilibrium was detected in 48 haplotypes containing 35 SNPs in 10 cancer driver genes. All together, 19 risk and eight protective alleles were identified in 23 out of 48 haplotypes. Homozygous individuals were mainly of European ancestry, whereas heterozygotes had at least one Native American and one African ancestry tract. Native-American ancestry for homozygous individuals with risk alleles for *HNF1B, CDH1*, and *BRCA1* was inferred for the first time. Results indicated that analysis of SNP polymorphism in the present admixed population has a high potential to identify new ancestry-associated alleles and haplotypes that modify cancer susceptibility differentially in distinct human populations. Future case-control studies with populations with a complex history of admixture could help elucidate ancestry-associated biological differences in cancer incidence and therapeutic outcomes.

## Introduction

Cancer is the second leading cause of death in developing countries, and its incidence is expected to increase by 75% by 2030 because of risk-associated lifestyle behaviors and the aging of the world’s population (Bray et al., 2012; Cai and Liu, 2019; Torre et al., 2015). In Brazil, as in other developing countries, the oldest old, those aged ≥80 years, is a rapidly growing population (Shetty, 2012; Mathers et al., 2015; Neumann and Albert, 2018). In 2020, the longevous elderly population is estimated to account for 2% of the Brazilian population, that is, 4,441,000 individuals. The oldest-old represent an adequate model of human longevity to study the adverse effects of progressive aging on cancer (Nolen et al., 2017). Longevous individuals present genetic variants associated with cancer susceptibility, and their phenotype manifestation might depend on their ancestry (Aizer et al., 2014; Jin et al., 2016; Özdemir and Dotto, 2017). It is well-established in literature that not only socio-economic but also biological differences can contribute to distinct cancer susceptibilities among human populations. Despite a comparable socio-economic status and lifestyle, Hispanics and Asians in the USA have an overall significantly decreased cancer susceptibility compared to Afro-Americans (Özdemir and Dotto, 2017). Especially the incidence of prostate cancer and triple-negative breast cancer (TNBC) are significantly increased among Afro-American men and women if compared to other populations (Newman et al., 2017, Jiagge et al., 2018; Lewis and Cropp, 2020). The hormone receptor positive subtypes of breast cancer in contrast, are more common among women of European origin compared to Afro-American women (Agboola et al., 2012; Newman *et al.*, 2019) 

Germline mutations in tumor suppressor genes, oncogenes, and DNA repair genes have been extensively investigated in genome-wide association studies (GWAS) using European populations (Haiman and Stram, 2010; Park et al., 2018). Cancer prediction based on genomic data frequently uses polygenic-risk scores; however, the predictive ability is lower for populations of different ancestry than those of European descent (Martin et al., 2019). Only 1% of cancer GWAS are performed in African and Latin American populations (Bodian et al., 2014; Fernandes et al., 2016; Park *et al.*, 2018). Genomic variants associated with cancer are often characterized by an ancestry-specific effect called “flip-flop”, in which variants associated with cancer in one ancestral population may have no or the opposite association as a result of linkage and epistatic effects (Wang et al., 2018). 

Comparative studies of distinct human populations have revealed differences in mutated allele frequency in cancer driver genes, including oncogenes and tumor suppressor genes (Özdemir and Dotto, 2017; Nakshatri et al., 2019; Bandlamudi and Taylor, 2020; Carrot-Zhang et al., 2020). For example, a South American case-control study identified 13 polymorphisms in a Colombian population that modified the risk of breast cancer, whereas an increase in the proportion of Native Americans decreased the risk of disease (Torres et al., 2019). Local ancestry inference has been used to increase the potential of GWAS through admixture-mapping analysis in ancestrally- diverse populations (Freedman et al., 2006; Yang et al., 2011). Up to date, few studies have inferred local ancestry for cancer causative mutations or identified novel ancestry-associated molecular features (Pasaniuc et al., 2011, Carrot-Zhang et al., 2020; Dutil et al., 2019; Ostrom et al., 2020; Yuan et al., 2018). Ancestry studies of polymorphisms that modify the risk of cancer in admixed populations may help identify genetic differences between populations. It is of special interest that recent studies identified genetic polymorphisms that might have suffered positive selection in native populations and at the same time modify cancer risk (Voskarides, 2018). Data indicated that specific alleles of the *PHD2* gene that are beneficial to hypoxic adaptation also increased the risk of lung cancer among Tibetans (Amorim et al., 2017). The *FADS1* and *FADS2* genes that are involved in fatty acid metabolism and are adaptive for a lipid-rich diet of Siberian Eskimos and Inuit were also suspected to increase the risk of colorectal cancer (Voskarides, 2018). This indicates that ancestry-related genetic polymorphisms can help to elucidate the differences of cancer susceptibility in distinct human populations. 

Because the locus-specific ancestry of cancer genomic variants in diverse populations remains unknown, in this study, we inferred the local ancestry of known cancer-associated single nucleotide polymorphisms (SNPs) and haplotypes in a highly admixed population of longevous individuals from Northeast Brazil. This region was chosen because of its high levels of admixture among European settlers, Native Americans, and enslaved Africans (Salzano and Sans, 2014; Moura et al., 2015; Mychaleckyj et al., 2017; de Farias et al., 2018) and increased, as well as a high prevalence of consanguineous marriages compared with other regions of Brazil (Weller et al., 2012).

We analyzed 35 cancer-associated SNPs of 10 genes, and performed a systematic review of the literature to identify the risk and protective alleles of haplotypes. The aim of the study was to identify new haplotypes harboring cancer-associated alleles and their corresponding ancestries. For this purpose, we investigated the frequency of different ancestries in haplotypes with alleles that may modify the risk of cancer and its etiology. A healthy elderly population of individuals aged ≥80 years without a history of cancer was analyzed to determine the proportions of protective and risk alleles in their haplotypes. 

## Material and Methods

### Study population

This cross-sectional study used population genomics methods to analyze 73 unrelated individuals, including 38 women and 35 men aged 80-102 years.

None of the participants was diagnosed with cancer during or before sampling. The elderly samples were obtained from the longitudinal cohort study “Health, Wellbeing and Aging” [“SABE project”] (Lebrão and Laurenti, 2005), which began in 2000 in São Paulo and was extended as a census-based study of elderly individuals aged >60 years from consanguineous communities in the Northeastern Brazil backlands. The “SABE - São Paulo” (SABE-SP) cohort comprises exomic variants of 609 elderly Brazilians available in ABraOM (Online Archive of Brazilian Mutations), a public variant repository (Naslavsky et al., 2017).

The samples were collected in the municipality of Brejo dos Santos in the backlands of the state of Paraíba, Brazil. This community is located at 360 km from Natal and 404 km from João Pessoa, which is at a considerable distance from these capitals of Rio Grande do Norte and Paraíba, respectively, both situated at the Atlantic coast. According to the Brazilian Institute of Geography and Statistics (IBGE), 876 (415 men and 461 women) of the 6198 inhabitants of Brejo dos Santos are >60-years-old. The present cohort represents approximately 10% of the total elderly (≥60-years-old) population of Brejo dos Santos. Of 878 unions in this community, 171 (19.48%) have a consanguineous background, resulting in a coefficient of endogamy of 0.00504 as reported previously (Weller et al., 2012).

The data sampling protocol and consent procedure were reviewed and approved by the National Committee for Ethics in Research (CONEP; Brazil) and are registered under protocol number 0359.0.133.000-11. Written informed consent was obtained from each participant. Consent to publish data anonymously was obtained from each participant.

### Selection of genes and corresponding SNPs

Cancer driver genes were identified from studies published between 2005 and 2020 using PubMed. The search terms used were “Oncogenes AND tumor suppressor genes”; and “Genes AND cancer”*.* Eligible studies were those reporting the frequency of cancer risk alleles, haplotypes associated with cancer risk, and ancestry. 

The following 20 genes were selected: the tumor suppressor genes *BRCA1*, *BRCA2*, *TP53*, *CDH1*, *ATM*, *MC1R*, *RB1*, and *VEGF* (Song et al., 2006; Al-Moundhri et al., 2010; Lahtz and Pfeifer, 2011; Zhao et al., 2012; Carraro et al., 2013; Tagliabue et al., 2018). The oncogenes *AURKA*, *CCND1*, *NCOA3*, *HNF1B*, *MMP7*, *MITF*, and *CDKN1A* (Burwinkel et al., 2005; Polistena et al., 2014; Hartman and Czyz, 2015; Yu et al., 2015; Vargas-Torres et al., 2016; Tang et al., 2017; Abel et al., 2018); and the DNA repair genes *XRCC1*, *ERCC1*, *ERCC2*, *ERCC5*, and *MLH1* (Xue et al., 2015; Meng et al., 2017).

### DNA extraction and genotyping

DNA was extracted from 5 mL of peripheral blood from each individual using the phenol-chloroform method. The quantity and quality of DNA were assessed by gel electrophoresis and spectrophotometry using Nanodrop ND-1000 (Thermo Scientific, Massachusetts, USA). The Axiom^®^ Genome-Wide LAT 1 Array (Affymetrix, USA) was used for genotyping the 73 longevous individuals following the manufacturer’s instructions. The array comprises 813,551 SNPs. 

Before the analysis, the raw archives of the 73 genotyped individuals were downloaded from Thermo Fisher cloud. SNPs were identified using the software Genotyping Console^TM^ (Version 4.2; Affymetrix Inc). The following filters were used for quality control: dish quality control ≥0.82, quality control call rate ≥92, average call rate for passing ≥97, and minor allele cut off ≥2. A SNP call rate >97% and a Hardy-Weinberg equilibrium of p >0.05 were applied. After filtering, 805,712 SNPs were included.

The minor allele frequency (MAF) of the “SABE Paraíba” (SABE-PB) population was compared with that of the SABE-SP population to determine the allele frequency distribution in two populations of long-lived elderly people living in different geographic regions. MAF information for the SABE-SP population was obtained from the Database of the Brazilian Online Archive of Mutations (ABraOM) (Naslavsky et al., 2017). 

The allelic frequency in diverse populations was estimated using the European (N = 1,006), African (N = 1,322), and admixed Native American (N = 694) reference populations from the deep catalog of human genetic variation - *1000 Genomes Project* obtained from the database of Single Nucleotide Polymorphisms using the GRCh37/hg19 reference assembly. The public database of human genetic variants and their relationship with human disease, *Clinvar*, was used to investigate the physical position (hg19), genetic function, classification, and clinical significance of SNPs (Landrum et al., 2016). 

Of the 805,712 SNPs, 2,948 were associated with cancer genes covered by the LAT array. Of these, 90 SNPs were selected for the analysis and classified based on the Ensembl genome browser predicted consequence as intronic variant, upstream and downstream variants, untranslated regions (3′-UTR; 5′-UTR), and synonymous or nonsynonymous variants. Cancer susceptibility variants were not identified for *MC1R, MLH1*, and *MITF* in the LAT array, and the remaining 17 genes were therefore included in the allele frequency analysis.

### Linkage disequilibrium (LD) of haplotypes and local ancestry inference

Haplotype block identification was performed by calculating the LD of each sequence combination for the 73 genotyped subjects using Haploview 4.0 software (Barrett et al., 2005). Haplotypes were filtered for ≥5% of the MAF. Haplotype analysis was performed by inferring LD blocks. The paired LD structure was built with all SNPs evaluated for each chromosome. Haplotypes that were considerably frequent or rare in the population had frequencies of >20% and <5%, respectively.

For local ancestry inference, the haplotypes of all genetic variants of the elderly and the 1000 genomes project (1KGP) database were analyzed using SHAPEIT2 software (Delaneau et al., 2013), and 1,092 samples from 1KGP Phase 3 were used as a reference panel (The 1000 Genomes Project Consortium *et al.*, 2015). The local ancestry inference (LAI) was estimated using the software RFMix (Maples et al., 2013)**.** We used 0.2 cM and two iterations of expectation maximization with the PopPhased option based on a standard forward-backward algorithm.

The 1KGP database, including Yoruba representing Africans, Iberians representing Europeans, and Peruvians representing admixed Native Americans, was used as described previously (de Farias et al., 2018). 

The SNP data were subjected to a cleaning process in which markers with >1% missing genotypes, large deviations from Hardy-Weinberg proportions (p ≤ 10^-8^), and MAF <0.01 were excluded, resulting in a final set of 667,855 SNPs after merging. The local ancestry inference was then performed using data from 328 individuals, including 73 from Brejo dos Santos (SABE-PB) and 255 from 1KGP. 

## Results

### Allele frequency

Of 90 selected SNPs (GRCh37 - hg19), 38 were located in intronic regions between exons and two were located in intergenic regions; three were non-coding transcript exon variants; there were eight synonymous and 21 non-synonymous (missense) substitutions; two were nonsense (stop gain), three downstream, six upstream, three in the 5′-UTR, and four in the 3′-UTR (Table S1). Of the 90 genetic variants associated with cancer susceptibility found in the Brejo dos Santos population, 60 had alleles identical to those described in the literature, seven had at least one allele in common, and 23 had different alleles that were not reported previously (Table S2).

The average MAF of the 90 SNPs was 27.7% (range, 0.68-47.94%) (SD = 0.139), whereas the frequency of the same 90 SNPs listed in 1KGP, was 36.96% (SD= 0.305), 30.63% (SD= 0.228), and 31.28% (SD= 0.2431) in populations of African, Caucasian, and Native American ancestry, respectively (Table 1; Table S2). A summary of the protective and risk cancer-associated alleles is shown in Text S1. 

**Table 1 -:** Frequencies (%) of homozygous genotypes in haplotypes with equal ancestry.

Gene	European	African	Native American
*BRCA1*	28.77 - 32.88	1.34 - 4.11	2.74
*CDH1*	31.51 - 41.53	-	2.74
*TP53*	24.66 - 47.95	4.11 - 5.48	-
*VEGF*	38.36 - 54.79	2.74 - 6.85	-
*HFN1B*	32.88 - 38.36	1.37 - 2.74	2.74
*MMP7*	34.25 - 35.65	-	-
*XRCC1*	31.51 - 35.62	4.11 - 6.85	-
*ERCC1/ERCC2/ERCC5*	28.77 - 58.90	2.74 - 6.85	-

### Local ancestry inference and ancestral haplotype lengths

In the Brejo dos Santos population, haplotypes containing protective or high-risk alleles were predominantly of European ancestry (Figure S1). For the *HNFB1* gene, 47 (64.38%) haplotypes were of European ancestry, 14 (19.17%) European / African, six (8.21%) European / admixed Native American, two haplotypes (2.73%) African and two (2.73%) African / admixed Native American, one haplotype (1.36%) European / Unknown and one (1.36%) African / Unknown. For the *BRCA1* gene, 37 (50.68%) haplotypes were of European descent, 22 (30.13%) European / African, five (6.84%) European / admixed Native American, five haplotypes (6.84%) were Africans, two (2.73%) African / admixed Native American and one haplotype (1.36%) of admixed Native American ancestry.

The individuals with homologous haplotypes of two different ancestries showed at least one tract of Native American or African origin. The combination of haplotypes with different ancestries was observed with high frequency, and some of them contained homozygous genotypes.

For the tumor suppressor gene *BRCA1*, the frequency of homologous haplotypes with only one ancestry was 28.77-32.88% for European and 1.34-4.11% for African ancestry, and 2.74% were of Native American ancestry (Table 1). Table 1 shows the range of frequencies of homozygous genotypes within homologous haplotypes with the same ancestry.

The average ancestry tract length was 9.12-81.71 megabases (MB) (Table S3). Longer tracts were observed for populations of European ancestry (30.49-81.71 MB), and variable shorter lengths were observed for African (10.48-26.63 MB) and Native American (9.1-44.94 MB) populations. One of the three ancestries was present in at least one haplotype among ten genes from elderly genotyped data. The average ancestral tract length of *VEGF* for Native American population was almost one half (44.94 MB) of that for the European population (81.71 MB), whereas for *BRCA1*, the African (26.63 MB) stretches were longer. The genes on chromosome 19 showed similar average sizes. Only *BRCA1* had a haplotype breakdown on continuous ancestry considering the four SNPs located close and within the gene (Table S3).

### Linkage disequilibrium and alleles of haplotypes that modify cancer risk

In the linkage disequilibrium (LD) analysis, 35 SNPs showed high LD. On chromosome 6 (*VEGF* gene), two blocks of linkage disequilibrium were identified (Figure S2a), on chromosome 11 (*MMP7* gene), one block (Figure S2b), on chromosome 13 (*ERCC5* gene), one block (Figure S2c), on chromosome 16 (*CDH1* gene) three blocks (Figure S2d), on chromosome 17 (*P53*, *HNF1B* and *BRCA1* genes) four blocks (Figure S2e), and on chromosome 19 (*XRCC1*, *ERCC2* and *ERCC1* genes) three LD blocks (Figure S2f). On chromosome 20 no LD blocks were identified (*NCOA3* and *AURKA* genes) (Figure S2g).

Of the 21 haplotypes with strong negative LD (D < 0), three were in *BRCA1,* two in *TP53*, and six in *CDH1* (Table 2); four haplotypes corresponded to the *VEGF* tumor suppressor gene and four to the *HNF1B* oncogene. Each of the DNA repair genes *XRCC1* and *ERCC1* had one haplotype with negative LD*.* Of the haplotypes with strong positive LD (D > 0), 11 were in tumor suppressor genes: three in *BRCA1*, two in *TP53*, three in *CDH1*, and three in *VEGF*. Thirteen were in DNA repair genes (Table 2): four in *XRCC1,* three in *ERCC1,* three in *ERCC2,* and three in *ERCC5*. Three haplotypes with strong positive LD were in the *MMP7* oncogene.

**Table 2 -:** The frequency of haplotypes with strong linkage disequilibrium (D), their corresponding SNPs, and genetic locus are shown. The logarithm of the odds ratio of the probability (LOD), the correlation coefficient between loci (r2), and the confidence intervals (CI) are shown for each haplotype. In the SNP column the order of named loci from the top to the bottom corresponds to the order of alleles in the haplotype column from the left to the right hand side. Alleles of haplotypes that increased the risk of cancer in previous studies are highlighted in grey, whereas protective alleles are highlighted in grey and written in bold-type letters.

Genetic locus		Haplotype	SNP	D	LOD	r2	CI	References
6p.12	*VEGF*	CC (69.2%)	**rs1005230** **rs25648**	-0.97	28.18	0.279	0.89-1.0	**rs1005230** (Linhares *et al.*, 2018) **rs25648** (Song et al., 2019)
		TC (19.4%)		-0.97	28.18	0.279	0.89-1.0	
		TT (11.3%)		-0.97	28.18	0.279	0.89-1.0	
		CCG (50.1%)	**rs3025039** **rs3025040** **rs10434**	-0.97	79.85	0.842	0.93-1.0	**rs3025039** (Hou *et al.*, 2017; Wang et al., 2018; Song et al., 2019) **rs3025040** (Jeon *et al.*, 2014; Liu *et al.*, 2017) **rs10434** (Jeon *et al.*, 2014; Zhu *et al.*, 2015; Wang *et al.*, 2018)
		CCA (29.1%)		1	12.6	0.092	0.86-1.0	
		TTG (18.1%)		1	13.93	0.105	0.88-1.0	
		CTG (2,3%)		1	13.93	0.105	0.88-1.0	
11p.13	MMP7	TT (53,8%)	**rs12285347** **rs11568818**	1	121.84	0.901	0.98-1.0	**rs12285347** (Hoffmann *et al.*, 2017) Increased PSA **rs11568818** (Beeghly-Fadiel *et al.*, 2009; Sharma *et al.*, 2012; Wu *et al.*, 2013; Wieczorek *et al.*, 2014; Kesh *et al.*, 2015; Horvat *et al.*, 2017; Xie *et al.*, 2016; Bialkowska *et al.*, 2018)
		CC (43.6%)		1	121.84	0.901	0.98-1.0	
		CT (2.6%)		1	121.84	0.901	0.98-1.0	
13p.13	*ERCC5*	AC (68,3%)	**rs4150351** **rs4150360**	1	13.26	0.126	0.85-1.0	**rs4150351** (Barry *et al.*, 2012; Ma *et al.*, 2012) **rs4150360** (Song *et al.*, 2017). Gastrointestinal toxicity
		AT (26.2%)		1	13.26	0.126	0.85-1.0	
		CT (5.5%)		1	13.26	0.126	0.85-1.0	
16p.13	*CDH1*	GT (73,0%)	**rs8056538** **rs2113200**	1	101.74	0.895	0.97-1.0	**rs8056538** **rs2113200** (Beeghly-Fadiel *et al.*, 2010; Carvajal-Carmona *et al.*, 2011). Colorectal cancer risk
		AA (24.8%)		1	101.74	0.895	0.97-1.0	
		AT (2.1%)		1	101.74	0.895	0.97-1.0	
		CA (78.8%)	**rs12919719** **rs17715799**	-0.97	82.37	0.838	0.93-1.0	**rs12919719** (Beeghly-Fadiel *et al.*, 2010); **rs17715799** (Jia *et al.*, 2015; Geng *et al.*, 2018).
		GT (18.4%)		-0.97	82.37	0.838	0.93-1.0	
		CT (2.4%)		-0.97	82.37	0.838	0.93-1.0	
		GC (50.1%)	**rs7188750** **rs4783689**	-0.96	13.52	0.103	0.82-1.0	**rs7188750** (Beeghly-Fadiel *et al.*, 2010); **rs4783689** (Jia *et al.*, 2015; Geng *et al.*, 2018)
		G**T** (27.5%)		-0.96	13.52	0.103	0.82-1.0	
		AC (22.2%)		-0.96	13.52	0.103	0.82-1.0	
17p13	*TP53*	ATC (58.4%)	**rs12951053** **rs2909430** **rs1042522**	1	3.56	0.035	0.58-1.0	**rs12951053** (Mechanic et al., 2007; Ru *et al.*, 2015; Bilous *et al.*, 2016) **rs2909430** (Mechanic et al., 2007; Bilous *et al.*, 2017) **rs1042522** (Mechanic *et al.*, 2007; Ru *et al.*, 2015; Asai *et al.*, 2019; Fernández-Mateos *et al.*, 2019; Zhang *et al.*, 2019; Ozola *et al.*, 2019; Pouladi *et al.*, 2019; Elshazli *et al.*, 2020; Kamiza et al., 2020; Liu *et al.*, 2020)
		CTG (15.0%)		1	28.29	0.258	0.93-1.0	
		ACG (14.6%)		-0.97	25.27	0.258	0.89-1.0	
		ATG (11.0%)		-0.95	72.4	0.606	0.9-0.98	
	*HNF1B*	CGG (49.3%)	**rs7501939** **rs11651052** **rs11658063**	-0.95	72.4	0.606	0.9-0.98	**rs7501939** (Setiawan *et al.*, 2012; Chornokur, 2013a; Nikolić et al., 2014; Kristiansen *et al.*, 2015; Ríos-Tamayo *et al.*, 2016; Oh et al., 2017a; Tong et al., 2018) **rs11651052** (Painter *et al.*, 2015). Endometrial cancer risk **rs11658063** (Jones et al., 2019)
		CAG (10.8%)		-0.95	72.4	0.606	0.9-0.98	
		TA**C** (31.5%)		-0.96	79.96	0.688	0.92-0.99	
		TAG (6,8%)		-0.95	51.56	0.453	0.89-0.99	
	*BRCA1*	TG (69.2%)	**rs16942** **rs1799949**	-0.99	101.43	0.868	0.96-1.0	**rs16942** (Cox *et al.*, 2011; Heramb *et al.*, 2015; Sagna et al., 2019) **rs1799949** (Ricks-Santi *et al.*, 2017). Age at diagnosis
		CG (2.7%)		-0.99	101.43	0.868	0.96-1.0	
		CA (27.9%)		-0.99	101.43	0.868	0.96-1.0	
		GC (69.5%)	**rs4986764** **rs4986765**	1	56.42	0.526	0.96-1.0	**rs4986764** (Ma *et al.*, 2013; Ren *et al.*, 2013; Shi *et al.*, 2013; Oussalah *et al.*, 2017; Liu *et al.*, 2018) **rs4986765** (Oussalah *et al.*, 2017)
		AC (11.7%)		1	56.42	0.526	0.96-1.0	
		AT (18.8%)		1	56.42	0.526	0.96-1.0	
19p13	*XRCC1*	CTGC (33.5%)	**rs25487** **rs25486** **rs1799782** **rs762507**	-0.97	104.68	0.879	0.94-1.0	**rs25487** (Roberts *et al.*, 2011; Jin *et al.*, 2015; Zhu *et al.*, 2016; Alimu *et al.*, 2018; Smolarz and Romanowicz, 2018; Qiao *et al.*, 2018; Minina *et al.*, 2019; Liu *et al.*, 2019; Aboul Enein *et al.*, 2020; Cai *et al.*, 2020; Smolarz *et al.*, 2019) **rs25486** (Roberts *et al.*, 2011) **rs1799782** (Alimu *et al.*, 2018; Bashir *et al.*, 2018; Li *et al.*, 2018; Zhu *et al.*, 2018; Cai *et al.*, 2020) **rs762507** (Sacerdote *et al.*, 2013) Bladder cancer survival
		TCGC (28.0%)		1	4.67	0.036	0.67-1.0	
		CTGT (27.6%)		1	15.43	0.152	0.89-1.0	
		CTAC (8.2%)		1	4.99	0.039	0.69-1.0	
		CCGC (2.1%)		1	16.28	0.164	0.9-1.0	
	*ERCC2*	TG (78.2%)	**rs13181** **rs1052555**	1	59.91	0.622	0.96-1.0	**rs13181** (Dai *et al.*, 2019b; Fernández-Mateos *et al.*, 2019; Li *et al.*, 2019; Smolarz *et al.*, 2019; Balkan *et al.*, 2020; Tavares *et al.*, 2020; Salimzadeh *et al.*, 2020; Zhao *et al.*, 2019) **rs1052555** (Yang *et al.*, 2005; Li *et al.*, 2020)
		GG (7.0%)		1	59.91	0.622	0.96-1.0	
		GA (14.8%)		1	59.91	0.622	0.96-1.0	
	*ERCC1*	AGCT (45.3%)	**rs1046282** **rs2336219** **rs3212986** **rs3212980**	1	9.21	0.132	0.83-1.0	**rs1046282** (Yin *et al.*, 2013) **rs2336219** (Ricci *et al.*, 2010; Dai *et al.*, 2019a) **rs3212986** (He *et al.*, 2018; Chaszczewska-Markowska *et al.*, 2019; Gholami *et al.*, 2019; Yang *et al.*, 2019; Bao *et al.*, 2020; Grenda *et al.*, 2020) **rs3212980** (Yin *et al.*, 2013).
		AACT (21.5%)		-0.95	84.59	0.791	0.91-0.98	
		GGAG (28.4%)		1	94.38	0.829	0.97-1.0	
		GGCT (3.8%)		1	7.82	0.114	0.79-1.0	

A literature search revealed that 25 of the 48 discovered haplotypes did not contain any allele with a known cancer risk modification function (Table 2). The search identified 19 risk alleles in 16 haplotypes and eight protective alleles in eight haplotypes (Table 2). The haplotype ACG of the *TP53* gene contained one risk and one protective allele (Table 2). The haplotype TTG of the *VEGF* gene contained three risk alleles (Table 2). The haplotype CT of the CDH1 gene contained two risk alleles (Table 2). Of the 16 haplotypes with risk alleles, eight (50.0%) had positive LD and eight (50.0%) had negative LD (Table 2). Of the eight haplotypes with protective alleles, two (25.0%) had positive LD and six (75%) had negative LD (Table 2).

## Discussion

Data on ancestry-specific variation may improve our understanding of the cancer risk associated with tumor suppressor genes, oncogenes, and DNA repair genes in diverse populations. The present local ancestry results for alleles and haplotypes in elderly individuals identified 48 haplotypes with strong LD that were not previously reported in the literature and alleles that have not been described as risk modifiers. In addition, it identified Native-American and African haplotypes harboring potential cancer variants that were not described previously.

Despite the strong level of endogamy, the present population showed a high degree of admixture: for nine of 10 genes, homozygous haplotypes were present in more than one of the three ancestries. One possible explanation for this result is that many of the haplotypes identified had more than one ancestry. However, most of the genes had combinations of haplotypes of different ancestries, such as single African or European ancestry and combinations of African/European ancestry. This indicated that endogamic effects dominated the present population only after admixture, namely, during the colonization of this region in Northeast Brazil. 

To the best of our knowledge, the 48 haplotypes identified in this study have not been described previously in the literature. Their potential function in cancer remains unknown, especially for the 25 haplotypes that do not bear any known allele associated with the modification of cancer risk. All the study participants were ≥80-years-old and did not have a history of cancer, suggesting that the 48 haplotypes included many protective alleles and few risk alleles. However, contrary to this hypothesis, of eight haplotypes containing one protective allele each, six had strong negative LD, and eight of the 16 haplotypes containing at least one risk allele had strong positive LD. Despite these arguments, the present Brazilian elderly population could serve as a model to describe ancestry-specific variation of haplotypes in cancer driver genes of different populations.

The 72 individuals included in the present study lived under similar socioeconomic conditions within a small community, suggesting that they had similar health-related behaviors such as physical activity, diet, smoking, and consumption of alcohol (Medeiros, 2018). One could speculate that not-sampled individuals of the present population who had cancer, might have other haplotypes with more risk alleles, respectively a lower number of corresponding protective alleles at the analyzed gene loci. However, as we did not perform a case-control study it is impossible with present data to draw any conclusion regarding the potential of haplotypes and corresponding alleles to diminish risk of cancer in the present elderly population. 

The high degree of trihybrid ancestry admixture suggests that such a population is a good model for cancer ancestry studies aimed at detecting new haplotypes that represent combinations of polymorphisms with differential effects on the incidence, prognosis, and therapeutic outcome of cancer among human populations. One problem associated with population-specific differences in the etiology, incidence, and prognosis of cancer among individuals can be discriminating between socio-demographic, lifestyle-related factors, and biological differences according to molecular markers (Özdemir and Dotto, 2017). The tumorigenic effect of polymorphisms in cancer driver genes may be enhanced or activated by lifestyle-related risk factors that differ among populations. In this scenario, population-specific molecular differences and lifestyle-related differences are correlated and can lead to meaningful results regarding the molecular differences among populations. On the other hand, lifestyle-related risk factors that differ among populations can lead to the false-positive association of molecular differences that may have no effect on cancer incidence and etiology, or they may mask molecular differences that have distinct biological effects. Many molecular differences among human populations do not affect cancer etiology (Carrot-Zhang et al., 2020). A recent study reported that most molecular differences between African, Asian, and European cancer patients are not limited to tumors, and can be specific to healthy tissues without affecting cancer etiology (Carrot-Zhang *et al.*, 2020).These confounders affecting the identification of molecular cancer-specific differences can be drastically reduced if case-control studies are combined with analysis of ancestry in an admixed population with a relatively homogenous background regarding lifestyle-related risk factors. Particularly with regard to low penetrance polymorphisms, haplotypes could be advantageous over single SNPs in the following aspects: 1. the combination of alleles in a haplotype may have a stronger effect on cancer incidence and etiology; 2. haplotypes with strong LD that contain SNPs with unknown functions may have been under selective pressure and have a specific function; 3. haplotypes with strong LD can also be the result of genetic drift and endogamic effects, and the association of cancer with these evolutionary forces should be analyzed; and 4. haplotype analysis may lead to the identification of SNPs with new functions. In the present study, haplotypes combined SNPs without a known function with SNPs related to cancer risk and etiology.

The complex mosaic derived from the three ancestries revealed a diverse combination of genotypes and ancestries, which reflected the multiple origins of cancer-associated mutations. The *BRCA1* and *CDH1* tumor suppressor genes had a predominant European ancestry and a lower Native American frequency, whereas *TP53* and *VEGF* exhibited a lower African contribution. *BRCA1* is a well-studied gene with a known Native American ancestry based on patient origin and allele frequency studies (Liede et al., 2002; Weitzel et al., 2007), although its presence in haplotypes containing Native American homozygous mutations was described for the first time, which was also the case for *CHD1*. African *BRCA1*, *TP53*, and *VEGF* were found in Brazilian women among other ancestrally diverse populations (Bodian et al., 2014; Fernandes et al., 2016; Oak et al., 2020) and may have a protective effect (Wang et al., 2018). 

The *HFN1B* gene, which is associated with prostate cancer, may be ancestry-specific for European Americans and Latinos but not for African-Americans (Waters et al., 2009); the present elderly population presented the Native American haplotypes, which were homozygous. European *MMP7*, which contains cancer-associated mutations, was associated with prostate cancer in European populations in a previous GWAS (Cook et al., 2014). *XRCC1* is associated with breast cancer risk in Mexican admixed individuals, whereas European and African haplotypes did not show cancer-related mutations (Macías-Gómez et al., 2015). The *ERCC* family genes have been associated with esophageal cancer in European patients (Boldrin et al., 2019), and are responsible for the platinum resistance pathway based on cell lines from ancestrally diverse populations (Wheeler et al., 2013). 

The flip-flop phenomenon might explain the presence of homozygous individuals for the risk allele with both European and African/Native American haplotypes (Wang et al., 2018). We hypothesized that the flip-flop phenomenon protected longevous individuals. The association between protective alleles and local ancestry inference should be further investigated in Brazilian patients using our dataset as the control in a case control study.

The present study was based on 35 SNPs that can potentially modify the risk of cancer. A literature research revealed that only 10 of these 35 SNPs were described in studies of African or Afro-American populations (Mechanic et al., 2007; Chornokur et al., 2013b; Nikolić et al., 2014; Oh et al., 2017b; Oussalah et al., 2017; Tong et al., 2018; Jones et al., 2019; Sagna et al., 2019; Song et al., 2019; Kamiza et al., 2020), suggesting that most association studies including these SNPs focused on European and Asian populations. The present results showed that a high number of individuals were homozygous for risk alleles with Native American and African ancestry that may modify the risk of cancer. Interestingly, the *HNF1B, CDH1*, and *BRCA1* genes were homozygous for the risk allele that combines Native American, African, or European haplotypes with different frequencies. 

Ancestry tracts reconstructed the demographic history of the elderly population of Brejo dos Santos; the haplotypes were broken down to a smaller size over generations as recombination events occurred, and long ancestral tracts were younger than short ones (Pool and Nielsen, 2009; Leitwein et al., 2020). The average ancestry tract length reflected the history of Northeast Brazil, as Native American haplotypes were older than African and European haplotypes. One exception was *VEGF*, which showed a younger African than Native American tract. This may reflect the origin of the Native American haplotypes after the trans-Atlantic slave trade from West-Africa to the Northeast Brazilian coast. The age of the haplotypes containing cancer-associated mutations remains unknown, and its estimation may help identify Brazilian autochthonous and allochthonous mutations.

An intriguing hypothesis for Native Americans showed a high frequency of cancer mutations associated with low temperatures and high altitude environments where Athabascans and Inuit live (Voskarides, 2018). This “cancer-cold” hypothesis is based on antagonistic pleiotropy effects conferring fitness benefits for SNPs selected under warmer environments such as Brejo dos Santos municipality. More studies are required to assess the extent of this influence with a higher number of municipalities spread by Caatinga semiarid biome.

One important limitation of the present study was the low sample number. Because the number of SNPs for each gene was low, the study may have identified only a small proportion of haplotypes. The potential function of the 48 new haplotypes remains unknown, and we cannot exclude the possibility that endogamic effects and genetic drift generated strong LD of haplotypes without an evolutionary function or contribution to cancer etiology. Another is the use of Peruvians as population reference instead of a Native American from other databases due to incompatibility of coverage and depth with our chosen SNP array.

## Conclusions

The present study identified 48 new haplotypes with strong LD and distinct African, Native American, and European ancestry, of which 23 contained alleles that were previously shown to modify the risk of different types of cancer. The results suggested that novel ancestry-specific haplotypes may explain differences in cancer incidence among distinct populations. The present study is the first to identify Native-American ancestry for individuals homozygous for the *HNF1B, CDH1*, and *BRCA1* risk alleles. The results indicated that a high number of haplotypes with the potential to modify cancer risk were associated with African ancestry. African and Native American haplotypes might be associated with increased risk of cancer and also have protective roles. Case-control studies should be performed to elucidate the potential function of the identified haplotypes by comparing the genetic data of healthy controls with those of cancer patients. Studies in an admixed population may help identify haplotypes that contribute to differences in cancer incidence and prognosis in distinct human populations. 

## Supplementary Material

The following online material is available for this article:

Figure S1 - The local ancestry of chromosome 17 using RFMix software.

Figure S2 - Pairwise linkage disequilibrium (LD) graph for chromosomes 6, 11, 13, 16, 17, and 19 generated by Haploview.

Table S1 **-**
Frequencies of the minor and major alleles and corresponding location, genetic function (Ensembl), and clinical significance **(**Clinvar**)** of the 90 SNPs for the 73 genotyped samples.

Table S2 - Literature review of the 90 SNPs identified in the Brejo dos Santos population showing the SNPs with the same alleles, different alleles, or at least one common allele found in the literature.

Table S3 - The average ancestry tract lengths in megabases and the respective standard deviations.

Text S1 - Risk alleles and protective alleles of the 48 haplotypes found in the literature.
