# Supplementary material for: Haplotypes of single cancer driver genes and their local ancestry in a highly admixed long-lived population of Northeast Brazil
Source: Genet Mol Biol. 2022 Feb 2;45(1):e20210172. doi: 10.1590/1678-4685-GMB-2021-0172 (PMC8811751; doi:10.1590/1678-4685-GMB-2021-0172)
Supplement: Figure S1 - [file 1415-4757-GMB-45-1-e20210172-s1.pdf]

Genetics and Molecular Biology
https://doi.org/10.1590/1678-4685-GMB-2021-0172

Supplementary material to "Haplotypes of single cancer driver genes and their local ancestry in a highly admixed long-lived population of Northeast Brazil"

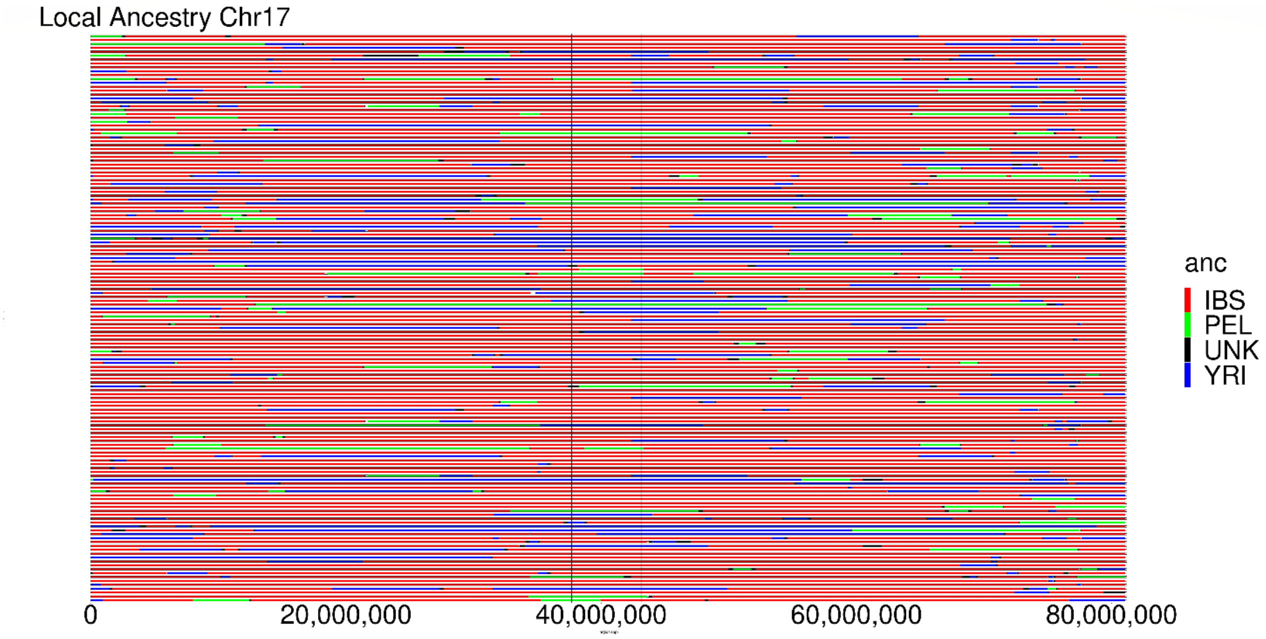

Figure S1 - The local ancestry of chromosome 17 using RFMix software. The figure represents the origin of the haplotypes for 73 individuals from Brejo dos Santos. Each individual has two lines of representation that refer to allele A and allele B. Vertical line 1 shows the *HNFB1* gene location whereas vertical line 2 represents the *BRCA1* gene position. Haplotypes for mixed individuals have been assigned to one of three possible ancestry: IBS [Europeans], YRI [Africans], PEL [admixed Native Americans]. UNK indicates "unknown" ancestry.
