# Supplementary material for: Haplotypes of single cancer driver genes and their local ancestry in a highly admixed long-lived population of Northeast Brazil
Source: Genet Mol Biol. 2022 Feb 2;45(1):e20210172. doi: 10.1590/1678-4685-GMB-2021-0172 (PMC8811751; doi:10.1590/1678-4685-GMB-2021-0172)
Supplement: Figure S2 - [file 1415-4757-GMB-45-1-e20210172-s2.pdf]

Genetics and Molecular Biology
https://doi.org/10.1590/1678-4685-GMB-2021-0172

Supplementary material to "Haplotypes of single cancer driver genes and their local ancestry in a highly admixed long-lived population of Northeast Brazil"

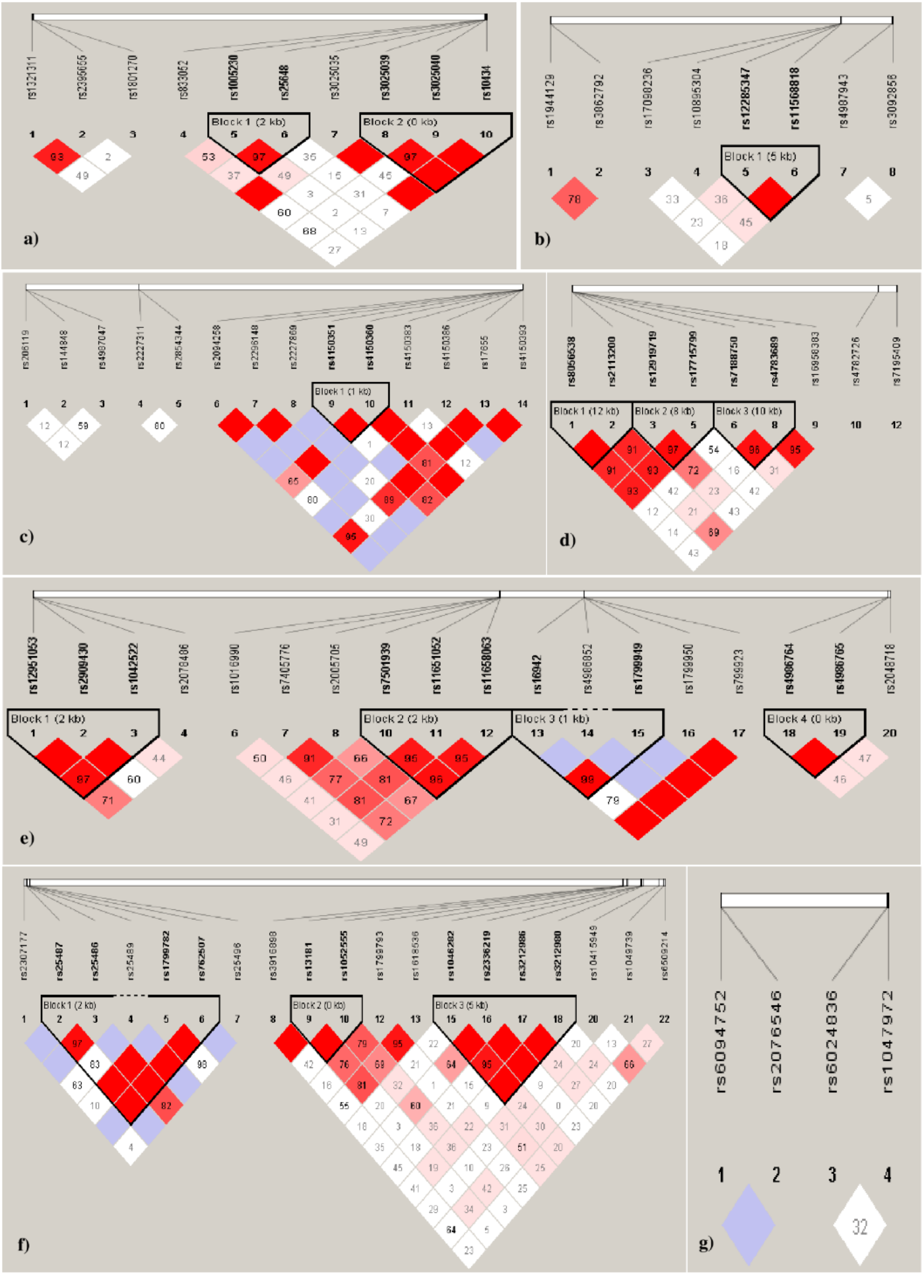

Figure S2 - Pairwise linkage disequilibrium (LD) graph for chromosomes 6, 11, 13, 16, 17, and 19 generated by Haploview. The red color shows high LD (D '= 1), indicating that two loci will be inherited by the next generation, along with a high association rate. The pink and white colors indicate low LD (D '<1). The number within each box indicates the D statistic value between the corresponding two SNPs. The length of each block shown in the graph is expressed in kilobases (kb). **a)** Chromosome 6 (*VEGF* gene), **b)** chromosome 11 (*MMP7* gene), **c)** chromosome 13 (*ERCC5* gene), **d)** chromosome 16 (*CDH1* gene), **e)** chromosome 17 (*P53*, *HNF1B* and *BRCA1* genes), **f)** chromosome 19 (*XRCC1*, *ERCC2* and *ERCC1* genes), **g)** chromosome 20 (*NCOA3* and *AURKA* genes).
