# Supplementary material for: Haplotypes of single cancer driver genes and their local ancestry in a highly admixed long-lived population of Northeast Brazil
Source: Genet Mol Biol. 2022 Feb 2;45(1):e20210172. doi: 10.1590/1678-4685-GMB-2021-0172 (PMC8811751; doi:10.1590/1678-4685-GMB-2021-0172)
Supplement: Table S1 - [file 1415-4757-GMB-45-1-e20210172-s3.pdf]

Genetics and Molecular Biology
https://doi.org/10.1590/1678-4685-GMB-2021-0172

Supplementary material to "Haplotypes of single cancer driver genes and their local ancestry in a highly admixed long-lived population of Northeast Brazil"

Table S1. Frequencies of the minor and major alleles and corresponding location, genetic function (Ensembl), and clinical significance **(**Clinvar**)** of the 90 SNPs for the 73 genotyped samples.

Ch	Gene	Position (hg19)	SNP	Major allele		Minor allele		Function	Clinical significance
					**F**		**F**		
17	*BRCA1*	41244000	rs16942	T	0.7054	C	0.2945	NS (Mi)	Benign
		41246481	rs1799950	T	0.9178	C	0.0821	NS (Mi)	Benign
		41244429	rs4986852	C	0.9931	T	0.0068	NS (Mi)	Benign
		41245466	rs1799949	G	0.7054	A	0.2945	S	Benign
		59763347	rs4986764	G	0.5684	A	0.4315	NS (Mi)	Benign
		59763465	rs4986765	T	0.6506	C	0.3493	S	PB
		59940819	rs2048718	C	0.5675	T	0.4315	5'UTR	PB
		41251931	rs799923	G	0.8151	A	0.1849	Intron	Benign
13	*BRCA2*	32914236	rs4987117	C	0.9863	T	0.0136	NS (Mi)	Benign
		32906729	rs144848	A	0.6917	C	0.3082	NS (Mi)	Benign
		32953529	rs4987047	A	0.9718	T	0.0281	NS (Mi)	Benign
		32889968	rs206119	G	0.7124	A	0.2876	Intron	Benign
17	*TP53*	7579472	rs1042522	G	0.6901	C	0.3098	NS (Mi)	PB
		7578645	rs2909430	C	0.8288	T	0.1712	Intron	Benign
		7583083	rs2078486	G	0.8699	A	0.1301	Intron	NR
		7592780	rs2287497	G	0.7362	A	0.2638	Intron	NR
		7577407	rs12951053	A	0.8836	C	0.1164	Intron	NR
20	*AURKA*	54961463	rs1047972	T	0.8356	C	0.1643	NS (Mi)	NR

Ch	Gene	Position (hg19)	SNP	Major allele		Minor allele		Function	Clinical significance
		54944068	rs6024836	G	0.6389	A	0.3611	Down	NR
11	*CCND1*	69462856	rs3862792	C	0.9305	T	0.0694	S	NR
		69453935	rs1944129	C	0.5206	T	0.4794	Up	NR
		69328764	rs614367	C	0.8836	T	0.1164	Up	NR
6	*CDKN1A*	36651971	rs1801270	C	0.8959	A	0.1041	NS (Mi)	Benign
		36645696	rs2395655	A	0.5417	G	0.4583	NS (Mi)	NR
		36622900	rs1321311	C	0.7946	A	0.2054	Intergen	NR
11	*ATM*	108121446	rs4987943	A	0.9862	G	0.0138	S	PB
		108159732	rs3092856	C	0.9864	T	0.0136	NS (Mi)	PB
19	*XRCC1*	44057574	rs1799782	G	0.9521	A	0.0479	NS (Mi)	NP
		44056412	rs25489	C	0.9453	T	0.0547	NS (Mi)	NP
		44055726	rs25487	T	0.6987	C	0.3013	NS (Mi)	NP
		44047825	rs2307177	T	0.9795	G	0.0205	NS (Mi)	NP
		44055898	rs25486	C	0.6806	T	0.3194	Intron	NP
		44058098	rs762507	T	0.6096	C	0.3904	Intron	NP
13	*ERCC5*	103528002	rs17655	G	0.6507	C	0.3493	NS (Mi)	Benign
		103515085	rs2227869	G	0.9247	C	0.0753	NS (Mi)	PB
		103527528	rs4150386	A	0.9237	C	0.0763	Intron	NP
		103496759	rs2094258	C	0.8288	T	0.1712	Intron	NP
		103498545	rs2296148	C	0.9042	T	0.0958	5’UTR	PB
		103522967	rs4150351	A	0.9521	C	0.0479	Intron	NP
		103524762	rs4150360	C	0.6929	T	0.3071	Intron	Benign
		103527230	rs4150383	G	0.7917	A	0.2083	Intron	NP
		103528658	rs4150393	A	0.9658	G	0.0342	Up	NP
6	*VEGF*	43736496	rs1005230	C	0.6439	T	0.3561	Up	NP

Ch	Gene	Position (hg19)	SNP	Major allele		Minor allele		Function	Clinical significance
		43738977	rs25648	C	0.9042	T	0.0958	S	NR
		43751359	rs3025035	C	0.8905	T	0.1095	NCtrans	NR
		43752536	rs3025039	C	0.7917	T	0.2083	3'UTR	NR
		43753212	rs10434	A	0.6781	G	0.3219	3'UTR	NR
		43753051	rs3025040	C	0.7877	T	0.2123	3'UTR	NR
		43723335	rs833052	C	0.9316	A	0.0684	Intergen	NR
11	*MMP7*	102387691	rs17098236	C	0.8836	T	0.1164	Down	NR
		102401661	rs11568818	T	0.5411	C	0.4589	Up	NR
		102389992	rs10895304	A	0.7362	G	0.2638	Down	NR
		102396607	rs12285347	C	0.5206	T	0.4794	Intron	NR
19	*ERCC1*	45910672	rs1046282	A	0.7570	G	0.2430	3'UTR	NR
		45912406	rs2336219	G	0.8768	A	0.1232	NS (Mi)	NR
		45916441	rs3212980	T	0.7946	G	0.2054	Intron	NR
		45981815	rs6509214	G	0.6850	T	0.3150	Intron	NR
		45962799	rs10415949	A	0.7398	G	0.2602	Intron	NR
		45923653	rs11615	A	0.5891	G	0.4109	S	NR
		45924362	rs3212948	G	0.5959	C	0.4041	Intron	NR
		45912736	rs3212986	C	0.7946	A	0.2054	SG	NR
19	*ERCC2*	45854919	rs13181	T	0.8288	G	0.1712	SG	NR
		45855524	rs1052555	G	0.8733	A	0.1267	S	Benign
		45857049	rs238416	T	0.5891	C	0.4109	Intron	NR
		45867259	rs1799793	C	0.8662	T	0.1338	NS (Mi)	NR
		45871606	rs1618536	T	0.5480	C	0.4520	Intron	NR
		45876967	rs11878644	T	0.6081	C	0.3819	Up	NR
13	*RB1*	48987032	rs2227311	A	0.8681	G	0.1319	5'UTR	NR

Ch	Gene	Position (hg19)	SNP	Major allele		Minor allele		Function	Clinical significance
		48997693	rs2854344	G	0.9316	A	0.0684	Intron	NR
17	*HNF1B*	36093022	rs7405776	G	0.6302	A	0.3698	Intron	NR
		36088915	rs1016990	G	0.5206	C	0.4794	NCTrans	NR
		36102381	rs11651052	G	0.8220	A	0.1780	Intron	NR
		36103872	rs11658063	G	0.5891	C	0.4109	Intron	NR
		36099840	rs11651755	T	0.5343	C	0.4657	Intron	NR
		36096300	rs2005705	G	0.8220	A	0.1780	Intron	NR
		36062299	rs3094509	G	0.7261	A	0.2739	Intron	NR
		36101156	rs7501939	C	0.6576	T	0.3424	Intron	NR
20	*NCOA3*	46268493	rs2076546	A	0.8699	G	0.1301	S	NR
		46256424	rs6094752	C	0.9384	T	0.061	NS (Mi)	NR
16	*CDH1*	68802282	rs8056538	G	0.8014	A	0.1986	Intron	NR
		83212398	rs8055236	G	0.7398	T	0.2602	Intron	NR
		83527592	rs7195409	G	0.7192	A	0.2808	Intron	NR
		68842895	rs7188750	G	0.8425	A	0.1575	NCTrans	NR
		68849837	rs6499199	C	0.9247	T	0.0753	Intron	NR
		68853671	rs4783689	C	0.6096	T	0.3904	Intron	NR
		82701333	rs4782726	A	0.7535	G	0.2465	Intron	NR
		68824008	rs4076177	A	0.6302	G	0.3698	Intron	NR
		68814948	rs2113200	T	0.8014	A	0.1986	Intron	NR
		68830511	rs17715799	A	0.8220	T	0.1780	Intron	NR
		68822341	rs12919719	C	0.8357	G	0.1643	Intron	NR

Abbreviations: 3’UTR = 3 prime UTR variant; 5’UTR = 5 prime UTR variant; Ch = Chromosome; Down = Downstream gene variant; F = Frequency; Intergen = Intergenic region variant; Intron = Intronic; Mi = Missense variant; NCTrans = non coding transcript exon variant; NP = Not provided; NR = Not reported; NS = Nonsynonymous substitution; PB = Probably benign; S = Synonymous substitution; SG = Stop gained variant; Up = Upstream gene variant.
