# Supplementary material for: Haplotypes of single cancer driver genes and their local ancestry in a highly admixed long-lived population of Northeast Brazil
Source: Genet Mol Biol. 2022 Feb 2;45(1):e20210172. doi: 10.1590/1678-4685-GMB-2021-0172 (PMC8811751; doi:10.1590/1678-4685-GMB-2021-0172)
Supplement: Table S2 - [file 1415-4757-GMB-45-1-e20210172-s4.pdf]

Genetics and Molecular Biology
https://doi.org/10.1590/1678-4685-GMB-2021-0172

Supplementary material to "Haplotypes of single cancer driver genes and their local ancestry in a highly admixed long-lived population of Northeast Brazil"

Table S2 - Literature review of the 90 SNPs identified in the Brejo dos Santos population showing the SNPs with the same alleles, different alleles, or at least one common allele found in the literature. The second allele always refers to the risk or protective allele.

Gene	SNP	Alleles	Classif.	Type of cancer	Population	Ref.
**SNPs with the same alleles**						
BRCA1	rs16942	T/C	Risk	Breast cancer	European / Asian/ North American	(Cox *et al.*, 2011)
	rs4986852	C/T	Risk	Breast cancer	European	(Romanowicz *et al.*, 2017)
	rs2048718	C/T	Protector	Cervical cancer	Asian	(Liu *et al.*, 2018)
	rs799923	G/A	Risk	Breast cancer	Asian	(Han *et al.*, 2017)
	rs1799949	G/A	Risk	Breast cancer	African-american	(Ricks-Santi *et al.*, 2017)
BRCA2	rs4987117	C/T	Risk	Breast cancer	European/Asian	(Solodskikh *et al.*, 2019)
	rs144848	A/C	Risk	Non-Hodgkin lymphoma and Breast cancer	Caucasian/Asian / African	(Li *et al.*, 2017)
			Protector	Lung cancer	Asian	(Lin *et al.*,2016)

Gene	SNP	Alleles	Classif.	Type of cancer	Population	Ref.
	rs4987047	A/T	Risk	Breast cancer	European	(Johnson *et al*., 2007)
P53	rs1042522	G/C	Risk	Head and neck cancer<br>Cervical cancer	European<br>Subsaharian population	(Fernández-Mateos *et al*., 2019)<br>(Kamiza *et al*., 2020)
	rs2078486	G/A	Risk	Ovarian cancer	Non hispanic	(Schildkraut *et al*., 2010)
AURKA	rs6024836	G/A	Risk	Breast cancer	Asian	(Ruan *et al*., 2011)
CCND1	rs3862792	C/T	Risk	Prostate cancer<br>Leukemia	European	(Rashkin *et al*, 2019)
	rs614367	C/T	Risk	Breast cancer	European	(Lambrechts *et al*., 2012)
CDKN1A	rs2395655	A/G	Risk	Esophageal carcinoma	Asian	(Yang *et al*., 2016)
ATM	rs3092856	C/T	Risk	Breast cancer	Asian	(Yue *et al*., 2018).
XRCC1	rs1799782	G/A	Risk	Breast cancer	Asian	(Alimu *et al*., 2018)
	rs25487	T/C	Protector	Bladder cancer	Asian	(Zhu *et al*., 2016)
	rs762507	T/C	Risk	Esophageal carcinoma	Asian	(Dai *et al*., 2019)
ERCC5	rs17655	G/C	Risk	Gastric cancer<br>Colorectal cancer	Asian /Caucasians	(Zhao *et al*., 2018)

Gene	SNP	Alleles	Classif.	Type of cancer	Population	Ref.
	rs2227869	G/C	Protector	Stomach cancer<br>Thyroid cancer	Asian<br>European	(Hussain *et al.*, 2009)<br>(Santos *et al.*, 2013)
	rs4150386	A/C	Risk	Endometrial cancer	American North	(Doherty *et al.*, 2011)
	rs2094258	C/T	Risk	Gastric cancer	Asian	(Zhang *et al.*, 2018)
	rs2296148	C/T	Risk	Prostate cancer	African-American	(Hooker *et al.*, 2008)
	rs4150351	A/C	Protector	Prostate cancer	North american	(Barry *et al.*,2012)
	rs4150383	G/A	Protector	Hepatocellular cancer	Asian	(Wang *et al.*, 2016)
	rs4150393	A/G	Risk	Squamous cell carcinoma of the head and neck	Non hispanic	(Ma *et al.*, 2012)
VEGF	rs1005230	C/T	Risk	Glioma and glioblastoma	Asian	(Linhares *et al.*, 2018)
	rs25648	C/T	Risk	Bladder cancer	European/ Asian	(Song *et al.*, 2019)
	rs3025035	C/T	Risk	Breast cancer	European	(Furriol *et al.*, 2015)
	rs3025039	C/T	Risk	Breast cancer	Asian	(Song *et al.*, 2019)
	rs10434	A/G	Risk	Gastric cancer	Asian	(Zhu *et al.*, 2015)
	rs3025040	C/T	Protector	Thyroid cancer	Asian	(Liu *et al.*, 2017)
	rs833052	C/A	Risk	Bladder cancer	Asian	(Yang *et al.*, 2014)

Gene	SNP	Alleles	Classif.	Type of cancer	Population	Ref.
MMP7	rs10895304	A/G	Risk	Breast cancer	Asian	(Beeghly-Fadiel *et al.*, 2008)
	rs12285347	C/T	Risk	Prostate cancer	American North	(Hoffmann *et al.*, 2017)
ERCC1	rs2336219	G/A	Risk	Colorectal cancer	Asian	(Dai *et al.*, 2015)
	rs6509214	G/T	Risk	Lung cancer	Asian	(Lee *et al.*, 2015)
	rs10415949	A/G	Risk	Lung cancer	Asian	(Lee *et al.*, 2015)
	rs3212948	G/C	Risk	Lung cancer	Asian	(Zhu *et al.*, 2014)
	rs3212986	C/A	Risk	Non-small cell lung cancer Pancreatic cancer	Asian	(Yu *et al.*, 2018)<br>(He *et al.*, 2016)
ERCC2	rs238416	T/C	Risk	Lung adenocarcinoma	Asian	(Han, 2017)
RB1	rs2854344	G/A	Protector	Breast cancer<br>Ovarian cancer	European	(Lesueur *et al.*, 2006)<br>(Song *et al.*, 2006)
HNFB1	rs7405776	G/A	Risk	Ovarian cancer	North american	(Shen *et al.*, 2013)
	rs1016990	G/C	Risk	Prostate cancer	European	(Berndt *et al.*, 2011)

Gene	SNP	Alleles	Classif.	Type of cancer	Population	Ref.
	rs11651052	G/A	Risco	Endometrial cancer	European	(Painter *et al.*, 2015)
	rs11658063	G/C	Protector	Lung cancer	African-american	(Jones *et al.*, 2019)
	rs11651755	T/C	Risk	Ovarian cancer	European	(Burghaus *et al.*, 2017)
	rs2005705	G/A	Risk	Endometrial cancer	Caucasian/Asian	(Painter *et al.*, 2015)
	rs7501939	C/T	Protector	Prostate cancer	European	(Nikolić *et al.*, 2014)
NCOA3	rs2076546	A/G	Protector	Breast cancer	European	(Burwinkel *et al.*, 2005)
	rs6094752	C/T	Risk	Breast cancer	Caucasian/ African-american	(Hartmaier *et al.*, 2009)
CDH1	rs8056538	G/A	Risk	Colorectal cancer	European	(Carvajal-Carmona *et al.*, 2011)
	rs7195409	G/A	Risk	Non-small cell lung cancer	Asian	(Li *et al.*, 2018)
	rs7188750	G/A	Protector	Breast cancer	Asian	(Beeghly-Fadiel *et al.*, 2008)
	rs6499199	C/T	Risk	Endometrial cancer	Asian	(Geng *et al.*, 2018)
	rs4783689	C/T	Protector	Endometrial cancer	China	(Geng *et al.*, 2018)
	rs4782726	A/G	Risk	Prostate cancer	African descent	(Chang *et al.*, 2011)

Gene	SNP	Alleles	Classif.	Type of cancer	Population	Ref.
	rs2113200	T/A	Risk	Colorectal cancer	European	(Carvajal-Carmona *et al.*, 2011)
	rs17715799	A/T	Risk	Endometrial cancer	Asian	(Geng *et al.*, 2018)
	rs12919719	C/G	Risk	Breast cancer	Asian	(Beeghly-Fadiel *et al.*, 2008)
**SNPs with different alleles**						
BRCA1	rs1799950	T/C	Protector	Breast cancer	Caucasian	(Xu *et al.*, 2018)
	rs4986764	G/A	Protector	Breast cancer	Asian	(Ma *et al.*, 2019)
	rs4986765	T/C	Risk	Breast cancer	Asian	(Ma *et al*, 2019)
BRCA2	rs206119	G/A	Risk	Nasopharyngeal carcinoma	Asian	(Qin *et al.*, 2011)
P53	rs2909430	C/T	Protector<br>Risk	Breast cancer<br>Leukemia	American North<br>European	(Sprague *et al.*, 2007)<br>(Bilous *et a*l., 2017)
AURKA	rs1047972	T/C	Protector<br>Risk	Breast cancer<br>Gastric cancer	Caucasian<br>European	(Tang *et al.*, 2014)<br>(Mesic *et al.*, 2019)
CCND1	rs1944129	C/T	Risk	Kidney cancer	Asian	(Xue *et al.*,2017)
CDKN1A	rs1321311	C/A	Risk	Esophageal carcinoma	Asian	(Geng *et al.*, 2015)

Gene	SNP	Alleles	Classif.	Type of cancer	Population	Ref.
XRCC1	rs25489	C/T	Risk<br>Risk	Breast cancer<br>Pancreatic cancer	European/Asian<br>North american	(Loizidou *et al.*, 2008)<br>(Chen *et al.*, 2019)
	rs25486	C/T	Risk<br>Risk	Breast cancer<br>Lung cancer	North american<br>Latinos	(Roberts *et al.*, 2011)<br>(Chang *et al.*, 2009)
ERCC5	rs4150360	C/T	Risk	Lung cancer	Asian	(Song *et al.*, 2017)
MMP7	rs17098236	C/T	Risk	Ovarian cancer	Non hispanic	(Johnatty *et al.*, 2010)
	rs11568818	T/C	Risk<br>Risk	Prostate cancer<br>Gastric cancer	American North<br>Asian	(Bialkowska *et al.*, 2018)<br>(Zare *et al.*, 2019)
ERCC1	rs1046282	A/G	Risk	Hepatocelular Carcinoma	Asian	(Huang *et al.*, 2019)
	rs3212980	T/G	Risk<br>Risk	Lung cancer<br>Ovarian cancer	Asian	(Yin *et a*l, 2013)<br>(Bao *et a*l., 2020)
	rs11615	A/G	Risk	Breast cancer	Asian	(Li *et al.*, 2018)
ERCC2	rs13181	T/G	Risk<br>Risk	Lung cancer<br>Pancreatic cancer	Caucasian<br>Asian	(Wu and Ding, 2014)<br>(Yang *et al.*, 2016)
	rs1052555	G/A	Risk - worst survival	Colorectal cancer	Asian	(Li *et al.*, 2020)

Gene	SNP	Alleles	Classif.	Type of cancer	Population	Ref.
	rs1799793	C/T	Risk	Gastric cancer<br>Esophageal carcinoma	Asian<br>Cucasian	(Li *et al.*, 2018)<br>(Boldrin *et al.*, 2019)
	rs11878644	T/C	Risk	Lung cancer	Latinos/ African-american	(Chang *et al.*, 2008)
RB1	rs2227311	A/G	Protector	Ovarian cancer	North american European	(Song *et al.*, 2006)
HNF1B	rs3094509	G/A	Risk	Prostate cancer	Asian	(Berndt *et al.*, 2011)
CDH1	rs4076177	A/G	Risk	Prostate cancer	European	(González *et al.*, 2003)
**SNPs with a common allele**						
P53	rs2287497	G/A	Risk	Ovarian cancer	European	(Mędrek *et al.*, 2013)
	rs12951053	A/C	Risk	Rectal cancer	Asian	(Zhang *et al.*, 2019)
CDKN1A	rs1801270	C/A	Protector	Cervical cancer	Asian	(Wang *et al.*, 2012)
ATM	rs4987943	A/G	Protector	Breast cancer	American	(Concannon *et al.*, 2008)
XRCC1	rs2307177	T/G	Risk	Head and neck cancer	Asian	(Mahjabeen *et al.*, 2013)

**Gene**	**SNP**	**Alleles**	**Classif.**	**Type of cancer**	**Population**	**Ref.**
ERCC2	rs1618536	T/C	Risk	Esophageal squamous cell carcinoma	Asian	(Zhang *et al.*, 2014)
CDH1	rs8055236	G/T	Risk	Prostate cancer	Asian	(Oh *et al.*, 2015)

References

Bao Y, Yang B, Zhao J, Shen S and Gao J (2020) Role of common ERCC1 polymorphisms in cisplatin-resistant epithelial ovarian cancer patients: A study in Chinese cohort. Int J Immunogenet 47:443-453.

Barry KH, Koutros S, Andreotti G, Sandler DP, Burdette LA, Yeager M, Freeman LEB, Lubin JH, Ma X, Zheng T *et al.* (2012) Genetic variation in nucleotide excision repair pathway genes, pesticide exposure and prostate cancer risk. Carcinogenesis 33:331-337.

Beeghly-Fadiel A, Long JR, Gao YT, Li C, Qu S, Cai Q, Zheng Y, Ruan ZX, Levy SE, Deming SL *et al.* (2008) Common MMP-7 polymorphisms and breast cancer susceptibility: A multistage study of association and functionality. Cancer Res 68:6453-6459.

Berndt SI, Sampson J, Yeager M, Jacobs KB, Wang Z, Hutchinson A, Chung C, Orr N, Wacholder S, Chatterjee N *et al.* (2011) Large-scale fine mapping of the HNF1B locus and prostate cancer risk. Human Mol Genet 20:3322-3329.

Białkowska K, Marciniak W, Muszyńska M, Baszuk P, Gupta S, Jaworska-Bieniek K, Sukiennicki G, Durda K, Gromowski T, Prajzendanc K *et al.* (2018) Association of zinc level and polymorphism in MMP-7 gene with prostate cancer in Polish population. PLoS One 13:e0201065.

Bilous N, Abramenko I, Saenko V, Chumak A, Dyagil I, Martina Z and Kryachok I (2017) Clinical relevance of TP53 polymorphic genetic variations in chronic lymphocytic leukemia. Leuk Res 58:1-8.

Boldrin E, Malacrida S, Rumiato E, Battaglia G, Ruol A, Amadori A and Saggioro D (2019) Association between ERCC1 rs3212986 and ERCC2/XPD rs1799793 and OS in patients with advanced esophageal cancer. Front Oncol 9:85.

Burghaus S, Fasching PA, Häberle L, Rübner M, Büchner K, Blum S, Engel A, Ekici AB, Hartmann A, Hein A *et al.* (2017) Genetic risk factors for ovarian cancer and their role for endometriosis risk. Gynecol Oncol 145:142-147.

Burwinkel B, Wirtenberger M, Klaes R, Schmutzler RK, Grzybowska E, Försti A, Frank B, Bermejo JL, Bugert P, Wappenschmidt B *et al.* (2005) Association of NCOA3 polymorphisms with breast cancer risk. Clin Cancer Res 11:2169-2174.

Carvajal-Carmona LG, Cazier JB, Jones AM, Howarth K, Broderick P, Pittman A, Dobbins S, Tenesa A, Farrington S, Prendergast J *et al.* (2011) Fine-mapping of colorectal cancer susceptibility loci at 8q23. 3, 16q22. 1 and 19q13.11: Refinement of association signals and use of in silico analysis to suggest functional variation and unexpected candidate target genes. Hum Mol Genet 20:2879-2888.

Chang BL, Spangler E, Gallagher S, Haiman CA, Henderson B, Isaacs W, Benford ML, Kidd LCR, Cooney K, Strom S *et al.* (2011) Validation of genome-wide prostate cancer associations in men of African descent. Cancer Epidemiol Biomarkers Prev 20:23-32.

Chang JS, Wrensch MR, Hansen HM, Sison JD, Aldrich MC, Quesenberry Jr CP, Seldin MF, Kelsey KT and Wiencke JK (2009) Base excision repair genes and risk of lung cancer among San Francisco Bay Area Latinos and African-Americans. Carcinogenesis 30:78-87.

Chang JS, Wrensch MR, Hansen HM, Sison JD, Aldrich MC, Quesenberry Jr CP, Seldin MF, Kelsey KT, Kittles RA, Silva G *et al.* (2008) Nucleotide excision repair genes and risk of lung cancer among San Francisco Bay Area Latinos and African Americans. Int J Cancer 123:2095-2104.

Chen J, Wang H and Li Z (2019) Association between polymorphisms of X-ray repair cross complementing group 1 gene and pancreatic cancer risk: a systematic review with meta-analysis. Pathol Oncol Res 25:897-904.

Concannon P, Haile RW, Børresen-Dale AL, Rosenstein BS, Gatti RA, Teraoka SN, Diep AT, Jansen L, Atencio DP, Langholz B *et al.* (2008). Variants in the ATM gene associated with a reduced risk of contralateral breast cancer. Cancer Res 68:6486-6491.

Cox DG, Simar J, Sinnett D, Hamdi Y, Soucy P, Ouimet M, Barjhoux L, Verny-Pierre C, McGuffog L, Healey S *et al.* (2011) Common variants of the BRCA1 wild-type allele modify the risk of breast cancer in BRCA1 mutation carriers. Hum Mol Genet 20:4732-4747.

Dai L, Tao H, Xiong G, Guan X, Bai Y and Xu X (2019) Association between intronic polymorphisms of XRCC1, ERCC2 and LIG1 genes and risk of esophageal squamous cell carcinoma in a Chinese Han population. Int J Clin Exp Med 12:2710-2719.

Dai Q, Luo H, Li XP, Huang J, Zhou TJ and Yang ZH (2015) XRCC1 and ERCC1 polymorphisms are related to susceptibility and survival of colorectal cancer in the Chinese population. Mutagenesis 30:441-449.

Doherty JA, Weiss NS, Fish S, Fan W, Loomis MM, Sakoda LC, Rossing MA, Zhao LP and Chen C (2011) Polymorphisms in nucleotide excision repair genes and endometrial cancer risk. Cancer Epidemiol Biomarkers Prev 20:1873-1882.

Fernández-Mateos J, Seijas-Tamayo R, Adansa Klain JC, Pastor Borgoñón M, Pérez-Ruiz E, Mesía R, Barco ED, Coloma CS, Dominguez AR, Daroqui JC *et al.* (2019) Genetic susceptibility in head and neck squamous cell carcinoma in a Spanish population. Cancers (Basel) 11:493.

Furriol J, Puntervoll HE, Knutsvik G, Mannelqvist M, Aziz S, Wik E and Akslen LA (2015) Associations between VEGF polymorphisms and clinical outcome in breast cancer. Cancer Res 75:135.

Geng TT, Xun XJ, Li S, Feng T, Wang LP, Jin TB and Hou P (2015) Association of colorectal cancer susceptibility variants with esophageal cancer in a Chinese population. World J Gastroenterol 21:6898-6904.

Geng YH, Wang ZF, Jia YM, Zheng LY, Chen L, Liu DG, Li XH, Tian XX and Fang WG (2018) Genetic polymorphisms in CDH1 are associated with endometrial carcinoma susceptibility among Chinese Han women. Oncol Lett 16:6868-6878.

González CA, Pera G, Agudo A, Palli D, Krogh V, Vineis P, Tumino R, Panico S, Berglund G, Simán H *et al.* (2003) Smoking and the risk of gastric cancer in the European Prospective Investigation Into Cancer and Nutrition (EPIC). Int J Cancer 107:629-634.

Han L (2017) A case-control study on the genetic risks for development of EGFR mutant and wild-type lung adenocarcinoma in never-smoking Chinese population. B. Sc. Thesis, University of Hong Kong, Pokfulam.

Han MR, Zheng W, Cai Q, Gao YT, Zheng Y, Bolla MK, Michailidou K, Dennis J, Wang Q, Dunning AM *et al.* (2017) Evaluating genetic variants associated with breast cancer risk in high and moderate-penetrance genes in Asians. Carcinogenesis 38:511-518.

Hartmaier RJ, Tchatchou S, Richter AS, Wang J, McGuire SE, Skaar TC, Rae JM, Hemminki K, Sutter C, Ditsch N *et al.* (2009) Nuclear receptor coregulator SNP discovery and impact on breast cancer risk. BMC Cancer 9:438.

He MG, Zheng K, Tan D and Wang ZX (2016) Association between ERCC1 and ERCC2 gene polymorphisms and susceptibility to pancreatic cancer. Genet Mol Res 15. DOI: 10.4238/gmr.15017879.

Hoffmann TJ, Passarelli MN, Graff RE, Emami NC, Sakoda LC, Jorgenson E, Habel LA, Shan J, Ranatunga DK, Quesenberry CP *et al.* (2017) Genome-wide association study of prostate-specific antigen levels identifies novel loci independent of prostate cancer. Nat Commun 8:14248.

Hooker S, Bonilla C, Akereyeni F, Ahaghotu C and Kittles RA (2008) NAT2 and NER genetic variants and sporadic prostate cancer susceptibility in African Americans. Prostate Cancer Prostatic Dis 11:349-356.

Huang YL, Wu JR, Fang M, Zhao HL, Liu ZM, Ye J, Huang LS, Zhu B (2019) The role of ERCC1 and AFP gene polymorphism in hepatocellular carcinoma. Medicine (Baltimore) 14:e15090.

Hussain SK, Mu LN, Cai L, Chang SC, Park SL, Oh SS, Wang Y, Goldstein BY, Ding BG, Jiang Q *et al.* (2009) Genetic variation in immune regulation and DNA repair pathways and stomach cancer in China. Cancer Epidemiol Biomarkers Prev 18:2304-2309.

Johnatty SE, Beesley J, Chen X, Macgregor S, Duffy DL, Spurdle AB, deFazio A, Gava N, Webb PM, Australian Ovarian Cancer Study Group *et al.* (2010) Evaluation of candidate stromal epithelial cross-talk genes identifies association between risk of serous ovarian cancer and TERT, a cancer susceptibility “hot-spot”. PLoS Genet 6:e1001016.

Johnson N, Fletcher O, Palles C, Rudd M, Webb E, Sellick G, Silva IS, McCormack V, Gibson L, Fraser A *et al.* (2007) Counting potentially functional variants in BRCA1, BRCA2 and ATM predicts breast cancer susceptibility. Hum Mol Genet 16:1051-1057.

Jones CC, Bradford Y, Amos CI, Blot WJ, Chanock SJ, Harris CC, Schwartz AG, Spitz MR, Wiencke JK, Wrensch MR *et al.* (2019) Cross-cancer pleiotropic associations with lung cancer risk in African Americans. Cancer Epidemiol Biomarkers Prev 28:715-723.

Kamiza AB, Kamiza S, Singini MG and Mathew CG (2020). Association of TP53 rs1042522 with cervical cancer in the sub-Saharan African population: A meta-analysis. Trop Med Int Health 25:666-672.

Lambrechts D, Truong T, Justenhoven C, Humphreys MK, Wang J, Hopper JL, Apicella C, Southey MC, Schmidt MK, Broeks A *et al.* (2012) 11q13 is a susceptibility locus for hormone receptor positive breast cancer. Hum Mutat 33:1123-1132.

Lee SY, Hong MJ, Jeon HS, Choi YY, Choi JE, Kang HG, Jung DK, Jin C, Do SK, Yoo SS *et al.* (2015) Functional intronic ERCC1 polymorphism from regulomeDB can predict survival in lung cancer after surgery. Oncotarget 6:24522-24532.

Lesueur F, Song H, Ahmed S, Luccarini C, Jordan C, Luben R, Easton DF, Dunning AM, Pharoah PD and Ponder BAJ (2006) Single-nucleotide polymorphisms in the RB1 gene and association with breast cancer in the British population. Br J Cancer 94:1921-1926.

Li B, Shi X, Yuan Y, Peng M, Jin H and Qin D (2018) ERCC1 rs11615 polymorphism increases susceptibility to breast cancer: a meta-analysis of 4547 individuals. Biosci Rep 38:BSR20180440.

Li Q, Guan R, Qiao Y, Liu C, He N, Zhang X, Jia X, Sun H, Yu J and Xu L (2017) Association between the BRCA2 rs144848 polymorphism and cancer susceptibility: A meta-analysis. Oncotarget 8:39818-39832.

Li YK, Xu Q, Sun LP, Gong YH, Jing JJ, Xing CZ and Yuan Y (2020) Nucleotide excision repair pathway gene polymorphisms are associated with risk and prognosis of colorectal cancer. World J Gastroenterol 26:307-323.

Lin Y, He F, Zhang X, Yu T, Liu Z and Cai L (2016) Polymorphism rs144848 in BRCA2 may reduce lung cancer risk in women: A case-control study in southeast China. Tumori 102:150-155.

Linhares P, Viana-Pereira M, Ferreira M, Amorim J, Nabiço R, Pinto F, Costa S, Vaz R and Reis RM (2018) Genetic variants of vascular endothelial growth factor predict risk and survival of gliomas. Tumour Biol 40:1010428318766273.

Liu D, Zheng Y, Wang M, Deng Y, Lin S, Zhou L, Yang P, Dai C, Xu P, Hao Q *et al.* (2018) Four common polymorphisms of BRIP1 (rs2048718, rs4988344, rs4986764, and rs6504074) and cancer risk: Evidence from 13,716 cancer patients and 15,590 cancer-free controls. Aging (Albany NY) 10:266-277.

Liu R, Ning L, Liu X, Zhang H, Yu Y, Zhang S, Rao W, Shi J, Sun H, and Yu Q (2017) Association between single nucleotide variants of vascular endothelial growth factor A and the risk of thyroid carcinoma and nodular goiter in a Han Chinese population. Oncotarget 8:15838-15845.

Loizidou MA, Michael T, Neuhausen SL, Newbold RF, Marcou Y, Kakouri E, Daniel M, Papadopoulos P, Malas S, Kyriacou K *et al.* (2008) Genetic polymorphisms in the DNA repair genes XRCC1, XRCC2 and XRCC3 and risk of breast cancer in Cyprus. Breast Cancer Res Treat 112:575-579.

Ma B, Wu J and Ma B (2019) Association between single nucleotide polymorphism of BRCA1-interacting protein C-terminal helicase 1 and early-onset breast cancer in Uygur and Han women in Xinjiang of China. Biotechnol Biotec Eq 33:1750-1756.

Ma H, Yu H, Liu Z, Wang LE, Sturgis EM and Wei Q (2012) Polymorphisms of XPG/ERCC5 and risk of squamous cell carcinoma of the head and neck. Pharmacogenet Genomics 22:50-57.

Mahjabeen I, Baig RM, Masood N, Sabir M, Inayat U, Malik FA and Kayani MA (2013) Genetic variations in XRCC1 gene in sporadic head and neck cancer (HNC) patients. Pathol Oncol Res 19:183-188.

Mędrek K, Magnowski P, Masojć B, Chudecka-Głaz A, Torbe B, Menkiszak J, Spaczyn´ski M, Gronwald J, Lubin´ski J and Górski B (2013) Association of common WRAP 53 variant with ovarian cancer risk in the Polish population. Mol Biol Rep 40:2145-2147.

Mesic A, Rogar M, Hudlez P, Bilalovic N, Eminovic I and Komel R (2019) Characterization and risk association of polymorphisms in Aurora kinases A, B and C with genetic susceptibility to gastric cancer development. BMC Cancer 19:919.

Nikolić ZZ, Branković AS, Savić-Pavićević DL, Preković SM, Vukotić VD, Cerović SJ, Filipović NN, Tomović SM, Romac SP and Brajušković GN (2014) Assessment of association between common variants at 17q12 and prostate cancer risk-evidence from Serbian population and meta-analysis. Clin Transl Sci 7:307-313.

Oh JJ, Park S, Lee SE, Hong SK, Lee S, Lee HM, Lee JK, Ho JN, Yoon S and Byun SS (2015) Genome-wide detection of allelic genetic variation to predict biochemical recurrence after radical prostatectomy among prostate cancer patients using an exome SNP chip. J Cancer Res Clin Oncol 141:1493-1501.

Painter JN, O'Mara TA, Batra J, Cheng T, Lose FA, Dennis J, Michailidou K, Tyrer JP, Ahmed S, Ferguson K *et al.* (2015) Fine-mapping of the HNF1B multicancer locus identifies candidate variants that mediate endometrial cancer risk. Hum Mol Genet 24:1478-1492.

Qin HD, Shugart YY, Bei JX, Pan QH, Chen L, Feng QS, Chen LZ, Huang W, Liu JJ, Jorgensen TJ *et al.* (2011). Comprehensive pathway-based association study of DNA repair gene variants and the risk of nasopharyngeal carcinoma. Cancer Res. 71:3000-3008

Rashkin SR, Graff RE, Kachuri L, Thai KK, Alexeeff SE, Blatchins MA, Cavazos TB, Corley DA, Emami NC, Hoffman JD *et al.* (2019) Pan-cancer study detects novel genetic risk variants and shared genetic basis in two large cohorts. Nat Commun 11:4423.

Ricks-Santi L, McDonald JT, Gold B, Dean M, Thompson N, Abbas M, Wilson B, Kanaan Y, Naab TJ and Dunston G (2017) Next generation sequencing reveals high prevalence of BRCA1 and BRCA2 variants of unknown significance in early-onset breast cancer in African American women. Ethn Dis 27:169-178.

Roberts MR, Shields PG, Ambrosone CB, Nie J, Marian C, Krishnan SS, Goerlitz DS, Modali R, Seddon M, Lehman T *et al.* (2011) Single-nucleotide polymorphisms in DNA repair genes and association with breast cancer risk in the web study. Carcinogenesis 32:1223-1230.

Romanowicz H, Strapagiel D, Słomka M, Sobalska-Kwapis M, Kępka E, Siewierska-Górska A, Zadrożny M, Bieńkiewicz J and Smolarz B (2017) New single nucleotide polymorphisms (SNPs) in homologous recombination repair genes detected by microarray analysis in Polish breast cancer patients. Clin Exp Med 17:541-546.

Ruan Y, Song AP, Wang H, Xie YT, Han JY, Sajdik C, Tian XX and Fang WG (2011) Genetic polymorphisms in AURKA and BRCA1 are associated with breast cancer susceptibility in a Chinese Han population. J Pathol 225:535-543.

Santos LS, Gomes BC, Gouveia R, Silva SN, Azevedo AP, Camacho V, Manita I, Gil OM, Ferreira TC, Limbert E *et al.* (2013) The role of CCNH Val270Ala (rs2230641) and other nucleotide excision repair polymorphisms in individual susceptibility to well-differentiated thyroid cancer. Oncol Rep 30:2458-2466.

Schildkraut JM, Iversen ES, Wilson MA, Clyde MA, Moorman PG, Palmieri RT, Whitaker R, Bentley RC, Marks JR and Berchuck A (2010) Association between DNA damage response and repair genes and risk of invasive serous ovarian cancer. PLoS One 5:e10061.

Shen H, Fridley BL, Song H, Lawrenson K, Cunningham JM, Ramus SJ, Cicek MS, Tyrer J, Stram D and Larson MC (2013) Epigenetic analysis leads to identification of HNF1B as a subtype-specific susceptibility gene for ovarian cancer. Nat Commun 4:1628.

Solodskikh SA, Panevina AV, Gryaznova MV, Gureev AP, Serzhantova OV, Mikhailov AA, Maslov AY and Popov VN (2019) Targeted sequencing to discover germline variants in the BRCA1 and BRCA2 genes in a Russian population and their association with breast cancer risk. Mutat Res 813:51-57.

Song Y, Yang Y, Liu L and Liu X (2019) Association between five polymorphisms in vascular endothelial growth factor gene and urinary bladder cancer risk: A systematic review and meta-analysis involving 6671 subjects. Gene 698:186–197.

Sprague BL, Trentham-Dietz A, Garcia-Closas M, Newcomb PA, Titus-Ernstoff L, Hampton JM, Chanock SJ, Haines JL and Egan KM (2007) Genetic variation in TP53 and risk of breast cancer in a population-based case-control study. Carcinogenesis 28:1680-1686.

Tang W, Qiu H, Jiang H, Wang L, Sun B and Gu H (2014) Aurora-A V57I (rs1047972) polymorphism and cancer susceptibility: A meta-analysis involving 27,269 subjects. PLoS One 9:e90328.

Wang B, Xu Q, Yang HW, Sun LP and Yuan Y (2016) The association of six polymorphisms of five genes involved in three steps of nucleotide excision repair pathways with hepatocellular cancer risk. Oncotarget 7:20357-20367.

Wang N, Wang S, Zhang Q, Lu Y, Wei H, Li W, Zhang S, Yin D and Ou Y (2012) Association of p21 SNPs and risk of cervical cancer among Chinese women. BMC Cancer 12:589.

Wu HY and Ding LY (2014) Comprehensive assessment of the association between XPD rs13181 polymorphism and lung cancer risk. Tumor Biol 35:8125-8132.

Xu GP, Zhao Q, Wang D, Xie WY, Zhang LJ, Zhou H, Chen SZ and Wu LF (2018) The association between BRCA1 gene polymorphism and cancer risk: A meta-analysis. Oncotarget 9:8681-8694.

Xue J, Qin Z, Li X, Zhang J, Zheng Y, Xu W, Cao Q and Wang Z (2017) Genetic polymorphisms in cyclin D1 are associated with risk of renal cell cancer in the Chinese population. Oncotarget 8:80889-80899.

Yang W, Li Y, Ning T, Cai H, Chen Z, Dong Y and Ke Y (2016) Polymorphisms in the 5′ upstream regulatory region of p21 WAF1/CIP1 and susceptibility to oesophageal squamous cell carcinoma. Sci Rep 6:22564.

Yang Y, Zhang X, Song D and Wei J (2014) Association between vascular endothelial growth factor gene polymorphisms and bladder cancer risk. Mol Clin Oncol 2:501-505.

Yin J, Vogel U, Wang H, Ma Y, Wang C, Liang D, Liu J, Li Yue, Zhao Y and Ma J (2013) HapMap-based study identifies risk sub-region on chromosome 19q13.3 in relation to lung cancer among Chinese. Cancer Epidemiol 37:923-929.

Yu T, Xue P, Cui S, Zhang L, Zhang G, Xiao M, Zheng X, Zhang Q, Cai Y, Jin C *et al.* (2018) Rs3212986 polymorphism, a possible biomarker to predict smoking-related lung cancer, alters DNA repair capacity via regulating ERCC1 expression. Cancer Med 7:6317-6330.

Yue LL, Wang FC, Zhang ML, Liu D, Chen P, Mei QB, Li PH, Pan HM and Zheng LH (2018) Association of ATM and BMI-1 genetic variation with breast cancer risk in Han Chinese. J Cell Mol Med 22:3671-3678.

Zare M, Jafari-Nedooshan J, Aghili K, Ahrar H, Jarahzadeh MH, Seifi-Shalamzari N, Zare-Shehneh M and Neamatzadeh H (2019) Association of MMP-7-181A> G polymorphism with colorectal cancer and gastric cancer susceptibility: A systematic review and meta-analysis. Arq Bras Cir Dig 32:e1449.

Zhang G, Xu Q, Wang Z, Sun L, Lv Z, Liu J, Xing C and Yuan Y (2019) p53 protein expression affected by TP53 polymorphism is associated with the biological behavior and prognosis of low rectal cancer. Oncol Lett 18:6807-6821.

Zhang Y, Wang L, Wang P, Song C, Wang K, Zhang J and Dai L (2014) Association of single nucleotide polymorphisms in ERCC2 gene and their haplotypes with esophageal squamous cell carcinoma. Tumor Biol 35:4225-4231.

Zhang Z, Yin J, Xu Q and Shi J (2018) Association between the XPG gene rs2094258 polymorphism and risk of gastric cancer. J Clin Lab Anal 32:e22564.

Zhao J, Chen S, Zhou H, Zhang T, Liu Y, He J, Zhu J and Ruan J (2018) *XPG* rs17655 G> C polymorphism associated with cancer risk: Evidence from 60 studies. Aging (Albany NY) 10:1073-1088.

Zhu G, Su H, Lu L, Guo H, Chen Z, Sun Z, Song R, Wang X, Li H and Wang Z (2016) Association of nineteen polymorphisms from seven DNA repair genes and the risk for bladder cancer in Gansu province of China. Oncotarget 7:31372-31383.

Zhu J, Hua RX, Jiang J, Zhao LQ, Sun X, Luan J, Lang Y, Sun Y, Shang K, Peng S *et al.* (2014) Association studies of ERCC1 polymorphisms with lung cancer susceptibility: A systematic review and meta-analysis. PLoS One 9:e97616.

Zhu LX, Ye XJ, Wang YG, Zhu JJ, Xie WZ, Zhao YM and Lai XY (2015) 3'-UTR polymorphism (rs10434) in the VEGF gene is associated with B-CLL in a Chinese population. Genet Mol Res 14:4085-4089.
