# Supplementary material for: Haplotypes of single cancer driver genes and their local ancestry in a highly admixed long-lived population of Northeast Brazil
Source: Genet Mol Biol. 2022 Feb 2;45(1):e20210172. doi: 10.1590/1678-4685-GMB-2021-0172 (PMC8811751; doi:10.1590/1678-4685-GMB-2021-0172)
Supplement: Table S3 - [file 1415-4757-GMB-45-1-e20210172-s5.pdf]

Genetics and Molecular Biology
https://doi.org/10.1590/1678-4685-GMB-2021-0172

Supplementary material to “Haplotypes of single cancer driver genes and their local ancestry in a highly admixed long-lived population of Northeast Brazil”

Table S3. The average ancestry tract lengths in megabases and the respective standard deviations.

**Ancestry**	***VEGF* (Chr6)**				
	rs1005230	rs25648	rs3025039	rs3025040	rs10434
European	81.71 (44.73)				
African	19.76 (15.33)				
Native-American	44.94 (36.85)				
UNK	31.06 (0)				

**Ancestry**	***MMP7* (Chr11)**	
	rs12285347	rs11568818
European	54.68 (35.40)	
African	23.34 (15.86)	
Native-American	16.60 (10.05)	
UNK	58.54 (0)	

**Ancestry**	***ERCC5* (Chr13)**	
	rs4150351	rs4150360
European	56.45 (31.53)	
African	19.92 (25.66)	
Native-American	19.01 (15.89)	
UNK	16.08 (0)	

**Ancestry**	***CDH1* (Chr. 16)**					
	rs8056538	rs2113200	rs12919719	rs17715799	rs7188750	rs4783689
European	56.44 (26.18)					
African	25.16 (29.94)					
Native-American	17.74 (11.78)					

**Ancestry**	***TP53* (Chr. 17)**		
	rs12951053	rs2909430	rs1042522
European	30.49 (27.30)		
African	10.48 (16.81)		
Native-American	9.12 (55.44)		
UNK	47.23 (0)		

**Ancestry**	***HNF1B* (Chr. 17)**		
	rs7501939	rs11651052	rs11658063
European	44.40 (24.15)		
African	26.12 (18.51)		
Native-American	19.88 (18.40)		

**Ancestry**	***BRCA1* (Chr. 17)**			
	rs16942	rs1799949	rs4986764	rs4986765
European	44.87 (24.22)		40.31 (25.21)	
African	26.63 (18.64)		18.11 (17.78)	
Native-American	18.99 (16.47)		21.37 (18.23)	
UNK	52.79 (0)		-	

Ancestry	*XRCC1* **(Chr. 19)**			
	rs25487	rs25486	rs1799782	rs762507
European	38.85 (17.67)			
African	24.26 (18.09)			
Native-American	14.90 (12.88)			
UNK	27.09 (88.78)			

Ancestry	*ERCC2* **(Chr. 19)**	
	rs13181	rs1052555
European	38.28 (17.93)	
African	22.58 (18.85)	
Native-American	11.55 (11.51)	
UNK	12.87 (0)	

Ancestry	***ERCC1 (Chr.19)***			
	rs1046282	rs2336219	rs3212986	rs3212980
European	38.28 (17.93)			
African	22.58 (18.85)			
Native-American	11.55 (11.51)			
UNK	12.87 (0)			
