# Supplementary material for: Haplotypes of single cancer driver genes and their local ancestry in a highly admixed long-lived population of Northeast Brazil
Source: Genet Mol Biol. 2022 Feb 2;45(1):e20210172. doi: 10.1590/1678-4685-GMB-2021-0172 (PMC8811751; doi:10.1590/1678-4685-GMB-2021-0172)
Supplement: Text S1 - [file 1415-4757-GMB-45-1-e20210172-s6.pdf]

Genetics and Molecular Biology
https://doi.org/10.1590/1678-4685-GMB-2021-0172

Supplementary material to "Haplotypes of single cancer driver genes and their local ancestry in a highly admixed long-lived population of Northeast Brazil"

Text S1. Risk alleles and protective alleles of the 48 haplotypes found in the literature.

The C allele of rs1005230 of the *VEGF* gene is associated with worse prognosis, but not with the risk of glioma in a Portuguese population (Linhares *et al.*, 2018). The T allele of rs25648 is associated with increased risk of bladder cancer in Asian and African populations (Song *et al.*, 2019). Meta-analysis revealed that the T allele of rs3025039 increases the risk of renal carcinoma and bladder cancer (Hou *et al.*, 2017; Song et al., 2019). Another meta-analysis including 32 case-control studies associated the T allele of rs3025039 and G allele of rs10434 with an increased risk of osteosarcoma (Wang *et al.*, 2018).

This was in contrast to a Chinese study showing that the A allele and the AA genotype of rs10434 increased the risk of B cell chronic lymphocytic leukemia (Zhu *et al.*, 2015). The T- allele of rs3025040 and G- allele of rs10434 increases the risk of colorectal cancer, as reported in a Korean study (Jeon et al., 2014). The TT genotype of rs3025040 increases the risk of papillary thyroid carcinoma (Liu *et al.*, 2017). In the present study the haplotype TTG of African/European ancestry and with a strong positive LD was composed of three risk alleles (Table 2).

The SNPs rs12285347 and rs11568818 of the *MMP7* gene shows a high correlation (r2=0.92) with prostate cancer in non-Hispanic whites (Hoffmann *et al.*, 2017). The GG genotype of rs11568818 is associated with decreased survival of breast cancer patients in the USA and advanced stage colorectal cancer in Slovenian patients (Beeghly-Fadiel *et al.*, 2009; Horvat *et al.*, 2017). A meta-analysis of 24 studies showed that the GG genotype increased the overall cancer risk in Asian populations (Wu *et al.*, 2013). The GG genotype was associated with increased risk of gallbladder cancer, as reported in Indian and Polish studies, and increased risk of gastric cancer in combination with tobacco addiction in India (Sharma *et al.*, 2012; Wieczorek *et al.*, 2014; Kesh *et al.*, 2015).

Furthermore, it was associated with increased risk of cervical cancer in a Han Chinese population and prostate cancer in a Polish population (Xie *et al.*, 2016; Bialkowska *et al.*, 2018).

The C allele and CC genotype of rs4150351 of the *ERCC5* gene are associated with decreased risk of squamous cell carcinoma and prostate cancer (Barry *et al.*, 2012; Ma *et al.*, 2012; Table 2), and rs4150360 was associated with gastrointestinal toxicity and the efficacy of platinum-based chemotherapy in non-small cell lung cancer patients in China (Song *et al.*, 2017).

The SNPs rs8056538 and rs2113200 of the *CDH1* gene are associated with colorectal cancer risk in European patients (Carvajal-Carmona *et al.*, 2011). The C allele of rs12919719 and the T allele of rs17715799 have been identified as risk alleles. The C- allele of rs12919719 was associated with increased risk of breast cancer in a Chinese population (Beeghly-Fadiel *et al.*, 2009). The T allele of rs17715799 was associated with increased risk of breast cancer and endometrial cancer in Chinese populations (Jia *et al.*, 2015; Geng *et al.*, 2018). Therefore, the haplotype CT was a combination of two risk alleles. The A allele of rs7188750 decreased the risk of breast cancer in a study from Shanghai (Beeghly-Fadiel *et al.*, 2009). In Chinese studies, the T allele and TT genotype of rs4783689 were associated with better prognosis of breast cancer patients and decreased risk of endometrial carcinoma (Jia *et al.*, 2015; Geng *et al.*, 2018).

Recent studies associated the risk of various types of cancer with the G allele of the SNP rs1042522 of the *TP53* gene: a revision of 59 case control studies originally performed in Asia, Europe, and the USA showed that the G allele increases the risk of colorectal cancer (Elshazli *et al.*, 2020; Table 2). The G allele was associated with increased risk of low rectal cancer and hepatoblastoma in Chinese populations (Zhang *et al.*, 2019; Liu *et al.*, 2020; Table 2). The G allele was associated with increased risk of gallbladder cancer, cutaneous melanoma, and breast cancer in populations of North India, Latvia, and Iran (Asai *et al.*, 2019; Ozola *et al.*, 2019; Pouladi *et al.*, 2019; Table 2). Furthermore, the G allele of rs1042522 was associated with increased risk of cervical cancer among women of an African, sub-Saharan population (Kamiza *et al.*, 2020; Table 2). Compared with CC, the GG genotype of rs1042522 increased the risk of head and neck squamous cell carcinoma in a Spanish cohort (Fernández-Mateos *et al.*, 2019).

The A allele of rs1042523 was identified as a protective allele against osteosarcoma: together with three other alleles of five SNPs, the A and G alleles of rs12951053 and rs1042522 reduced the risk of osteosarcoma in a Chinese population (Ru *et al.*, 2015; Table 2). Furthermore, the G allele of rs12951053, the A allele of rs2909430, and the G allele of rs1042522 in combination with two other alleles of SNPs increased the risk of lung cancer among African Americans (Mechanic *et al.*, 2007). In the present study, the alleles of the SNPs rs12951053 and rs2909430 were A or C and T or C, respectively, but never G (Table 2). The GG genotypes of rs2909430 and rs12951053 increased the risk of chronic lymphocytic leukemia in Europeans (Bilous *et al.*, 2017; Bilous *et al.*, 2016).

The C- allele of rs11658063 of the *HNF1B* gene protects against lung cancer in Afro-Americans (Jones *et al.*, 2019). Haplotypes containing the A allele of rs11651052 present endometrial cancer risk in Caucasians (Painter *et al.*, 2015). The G allele of rs7501939 modified the risk of cancer in previous studies: the G allele of rs7501939 increases the risk of prostate cancer in African American, European, and Asian populations (Chornokur *et al.*, 2013; Nikolić *et al.*, 2014; Oh *et al.*, 2017; Tong *et al.*, 2018). In European studies, it was associated with testicular germ cell tumor (Kristiansen *et al.*, 2015), endometrial cancer (Setiawan *et al.*, 2012), and poor overall survival of multiple myeloma patients (Ríos-Tamayo *et al.*, 2016).

In the *BRCA1* gene, the G allele of rs16942 is associated with breast and cervical cancer in African patients with a family history (Sagna *et al.*, 2019). The rare C allele of rs16942 is associated with decreased risk of breast cancer among French women (Cox *et al.*, 2011) and with early onset before 40 years of both types of cancer in a cohort of Norwegian women (Heramb *et al.*, 2015). The rs1799949 is associated with age at diagnosis of breast cancer in African American women (Ricks-Santi *et al.*, 2017).

The C allele of rs4986764 increases the risk of breast (Ren *et al.*, 2013) and cervical cancer in Chinese populations (Ma *et al.*, 2013). The T allele is associated with decreased risk of breast cancer among Europeans, but not among Asians (Shi *et al.*, 2013). Polymorphisms of rs4986764 are associated with decreased risk of cervical cancer (Liu *et al.*, 2018). The haplotype AAA of the minor alleles of rs4986763, rs4986764, and rs4986765 is associated with increased risk of hepatocellular carcinoma among persons with African ancestry (Oussalah *et al.*, 2017).

The T allele of rs25487 and the A allele of rs1799782 are associated with increased risk of laryngeal cancer in Chinese populations (Alimu *et al.*, 2018; Table 2). In studies performed in South Korea and China, the heterozygous CT genotype of rs25487 increased the risk of lymph node metastasis in gastric cancer and the risk of bladder cancer compared with the CC genotype (Jin *et al.*, 2015; Zhu *et al.*, 2016; Table 2).

For the *XRCC1* gene, the A allele of rs25487 is associated with hepatocellular carcinoma in patients from Egypt and China (Cai *et al.*, 2020; Aboul Enein *et al.*, 2020), increased risk of breast cancer in Chinese and European populations (Liu *et al.*, 2019; Smolarz *et al.*, 2019), endometrial cancer in a European population (Smolarz and Romanowicz, 2018), and in combination with polymorphisms in three other genes, with increased risk of lung cancer (Minina *et al.*, 2019). By contrast, G to A of rs25487 significantly reduces the risk of breast cancer in Mongoloid and Caucasoid populations (Qiao *et al.*, 2018).

Haplotypes of heterozygous and homozygous risk alleles of rs25486 and rs25487 are associated with increased risk of breast cancer among post-menopausal women in the USA (Roberts *et al.*, 2011). Polymorphisms of rs1799782 are associated with hepatocellular carcinoma in Chinese populations (Cai *et al.*, 2020; Table 2). The homozygous and heterozygous T alleles of rs1799782 are associated with increased risk of lymphatic breast cancer metastasis in Chinese patients (Li *et al.*, 2018; Table 2).

The T allele of rs1799782 is associated with increased risk of papillary thyroid carcinoma in a Chinese population (Zhu *et al.*, 2018). By contrast, the homozygous T allele of rs1799782 decreases the risk of thyroid cancer (Bashir *et al.*, 2018). The T allele of rs762507 is associated with the risk of esophageal squamous cell carcinomas related to gender in Chinese populations. A strong association was identified in the female subgroup (Dai L *et al.*, 2019).

The G allele of rs13181 of the *ERCC2* gene increases the risk of breast cancer and head and neck squamous cell carcinoma in European populations (Smolarz *et al.*, 2019; Fernández-Mateos *et al.*, 2019). The TT genotype of rs13181 is associated with increased risk of hepatocellular carcinoma and gastric cancer, as reported in a Turkish study (Balkan *et al.*, 2020). Polymorphisms of rs13181 were associated with pancreatic and lung cancer risk (Dai P *et al.*, 2019; Li *et al.*, 2019) and risk of

glioma (Tavares *et al.*, 2020). The C and T allele of rs13181 and rs1052555 modify the risk of several types of cancer: the C allele of rs13181 is associated with a significantly increased risk of colorectal cancer in a European population (Salimzadeh *et al.*, 2020).

In Chinese patients with hepatocellular carcinoma, overall survival was significantly longer for the rs13181 CC genotype than for the AA genotype (Zhao *et al.*, 2019). For rs1052555, A and G alleles were found in the population of the present study, but only C and T were identified as risk alleles: the C allele of rs1052555 increased risk and was also associated with poor survival of Chinese colorectal cancer patients (Li *et al.*, 2020). In a previous study performed in the USA, the T allele of rs1052555 was associated with oligodendrogliomas (Yang *et al.*, 2005).

The AA genotype of rs2336219 of the *ERCC1* gene is associated with increased risk of colorectal cancer in a Chinese population (Dai L *et al.*, 2019) and the T- allele has a protective effect against bladder cancer in an Italian population (Ricci-Vitiani *et al.*, 2010). The SNP rs3212986 had an A or C allele in the present haplotypes. On one hand, the A versus C allele of rs3212986 increased ovarian cancer susceptibility in a Chinese study (Yang *et al.*, 2019), and the CC genotype was associated with longer progression free survival among European lung cancer patients (Grenda *et al.*, 2020).

Furthermore, polymorphisms of rs3212986 are associated with cisplatin-resistant epithelial ovarian cancer in a Chinese cohort (Bao *et al.*, 2020). On the other hand, the AA genotype of rs3212986 is protective against gastric cancer in a Chinese population (He *et al.*, 2018). The other alleles of the present haplotypes were not described as risk or protective alleles in the literature. The T- allele of rs3212986 is associated with increased risk of colorectal cancer (Gholami *et al.*, 2019) and increased, within a haplotype of five SNPs, risk of lung cancer in Asian populations (Yin *et al.*, 2013). The GG genotype is associated with increased risk of lung cancer (Chaszczewska-Markowska *et al.*, 2019). The A allele of rs3212980 in a haplotype composed of four SNPs from different genes increases the risk of breast cancer (Yin *et al.*, 2013).

References

Aboul Enein AA, Khaled IAA, Khorshied MM, Abdel-Aziz AO, Zahran N, El Saeed AM, Shousha HI and Abdel Rahman HA (2020) Genetic variations in DNA-repair genes (XRCC1, 3, and 7) and the susceptibility to hepatocellular carcinoma in a cohort of Egyptians. J Med Virol 92:3609-3616.

Alimu N, Qukuerhan A, Wang S, Abdurehim Y, Kuyaxi P, Zhang B and Yasheng Y (2018) The association between XRCC1 polymorphism and laryngeal cancer susceptibility in different ethnic groups in Xinjiang, China. Int J Clin Exp Pathol 11:4595-4604.

Asai T, Tsuchiya Y, Mishra K, Behari A, Shukla P, Ikoma T, Kapoor VK and Nakamura K (2019) Carcinogen metabolism pathway and tumor suppressor gene polymorphisms and Gallbladder cancer risk in North Indians: A hospital-based case-control study. Asian Pac J Cancer Prev 20: 3643-3647.

Balkan E, Bilici M, Gundogdu B, Aksungur N, Kara A, Yasar E, Dogan H and Ozturk G (2020) ERCC2 Lys751Gln rs13181 and XRCC2 Arg188His rs3218536 Gene polymorphisms contribute to susceptibility of colon, gastric, liver, lung and prostate cancer. J BUON 25:574-581.

Bao Y, Yang B, Zhao J, Shen and Gao J (2020) Role of common ERCC1 polymorphisms in cisplatin-resistant epithelial ovarian cancer patients: A study in Chinese cohort. Int J Immunogenet 47:443-453.

Barry KH, Koutros S, Andreotti G, Sandler DP, Burdette LA, Yeager M, Freeman LEB, Lubin JH, Ma X, Zheng T *et al.* (2012) Genetic variation in nucleotide excision repair pathway genes, pesticide exposure and prostate cancer risk. Carcinogenesis 33:331-337.

Bashir K, Sarwar R, Fatima S, Saeed S, Mahjabeen I and Akhtar Kayani M (2018) Haplotype analysis of XRCC1 gene polymorphisms and the risk of thyroid carcinoma. J BUON 23:234-243.

Beeghly-Fadiel A, Lu W, Gao YT, Long J, Deming SL, Cai Q, Zheng Y, Shu XO and Zheng W (2010) E-cadherin polymorphisms and breast cancer susceptibility: A report from the Shanghai Breast Cancer Study. Breast Cancer Res Treat 121:445-452.

Beeghly-Fadiel A, Shu XO, Long J, Li C, Cai Q, Cai H, Gao YT and Zheng W (2009) Genetic polymorphisms in the MMP-7 gene and breast cancer survival. Int J Cancer 124:208-214.

Białkowska K, Marciniak W, Muszyńska M, Baszuk P, Gupta S, Jaworska-Bieniek K, Sukiennicki G, Durda K, Gromowski T, Prajzendanc K *et al.* (2018) Association of zinc level and polymorphism in MMP-7 gene with prostate cancer in Polish population. PLoS One 13:e0201065.

Bilous N, Abramenko I, Saenko V, Chumak A, Dyagil I, Martina Z and Kryachok I (2017) Clinical relevance of TP53 polymorphic genetic variations in chronic lymphocytic leukemia. Leuk Res 58:1-8.

Bilous NI, Abramenko IV, Chumak AA, Dyagil IS and Martina ZM (2016) The distribution of TP53 gene polymorphisms in chronic lymphocytic leukemia patients, sufferers of Chornobyl nuclear power plant accident. Exp Oncol 38:252–256.

Cai W, Liu X, Li Y, Bi B, Liu L and Wang Z (2020) New sights on the associations between the XRCC1 gene polymorphisms and hepatocellular carcinoma susceptibility. J Cell Biochem 121:1005-1022.

Carvajal-Carmona LG, Cazier JB, Jones AM, Howarth K, Broderick P, Pittman A, Dobbins S, Tenesa A, Farrington S, Prendergast J *et al.* (2011) Fine-mapping of colorectal cancer susceptibility loci at 8q23.3, 16q22.1 and 19q13.11: Refinement of association signals and use of in silico analysis to suggest functional variation and unexpected candidate target genes. Hum Mol Genet 20:2879-2888.

Chaszczewska-Markowska M, Kosacka M, Chryplewicz A, Dyła T, Brzecka A and Bogunia-Kubik K (2019) ECCR1 and NFKB2 polymorphisms as potential biomarkers of non-small cell lung cancer in a Polish population. Anticancer Res 39:3269-3272.

Chornokur G, Amankwah EK, Davis SN, Phelan CM, Park JY, Pow-Sang J and Kumar NB (2013) Variation in HNF1B and obesity may influence prostate cancer risk in African American men: A pilot study. Prostate Cancer 2013:384594.

Cox DG, Simard J, Sinnett D, Hamdi Y, Soucy P, Ouimet M, Barjhoux L, Verny-Pierre C, McGuffog L, Healey S *et al.* (2011) Common variants of the BRCA1 wild-type allele modify the risk of breast cancer in BRCA1 mutation carriers. Hum Mol Genet 20:4732-4747.

Dai L, Tao H, Xiong G, Guan X, Bai Y and Xu X (2019) Association between intronic polymorphisms of XRCC1, ERCC2 and LIG1 genes and risk of esophageal squamous cell carcinoma in a Chinese Han population. Int J Clin Exp Med 12:2710-2719.

Dai P, Li J, Li W, Qin X, Wu X, Di W and Zhang Y (2019) Genetic polymorphisms and pancreatic cancer risk: A PRISMA-compliant systematic review and meta-analysis. Medicine (Baltimore) 98:e16541.

Elshazli RM, Toraih EA, Elgaml A, Kandil E and Fawzy MS (2020) Genetic polymorphisms of TP53 (rs1042522) and MDM2 (rs2279744) and colorectal cancer risk: An updated meta-analysis based on 59 case-control studies. Gene 734:144391.

Fernández-Mateos J, Seijas-Tamayo R, Adansa Klain JC, Pastor Borgoñón M, Pérez-Ruiz E, Mesía R, del Barco E, Salvador Coloma C, Rueda Dominguez A, Caballero Daroqui J *et al.* (2019). Genetic susceptibility in head and neck squamous cell carcinoma in a Spanish population. Cancers (Basel) 11:493.

Geng YH, Wang ZF, Jia YM, Zheng LY, Chen L, Liu DG, Li XH, Tian XX and Fang WG (2018) Genetic polymorphisms in CDH1 are associated with endometrial carcinoma susceptibility among Chinese Han women. Oncol Lett 16:6868-6878.

Gholami M, Larijani B, Sharifi F, Hasani-Ranjbar S, Taslimi R, Bastami M, Atlasi R and Amoli MM (2019) MicroRNA-binding site polymorphisms and risk of colorectal cancer: A systematic review and meta-analysis. Cancer Med 8:7477-7499.

Grenda A, Błach J, Szczyrek M, Krawczyk P, Nicoś M, Kuźnar Kamińska B, Jakimiec M, Balicka G, Chmielewska I, Batura-Gabryel H *et al.* (2020) Promoter polymorphisms of TOP2A and ERCC1 genes as predictive factors for chemotherapy in non-small cell lung cancer patients. Cancer Med 9:605-614.

He J, Zhuo ZJ, Zhang A, Zhu J, Hua RX, Xue WQ, Zhang HD, Zhang JB, Li XZ and Jia WH (2018) Genetic variants in the nucleotide excision repair pathway genes and gastric cancer susceptibility in a southern Chinese population. Cancer Manag Res 10:765-774.

Heramb C, Ekstrøm PO, Tharmaratnam K, Hovig E, Møller P and Mæhle L (2015) Ten modifiers of BRCA1 penetrance validated in a Norwegian series. Hered Cancer Clin Pract 13:14.

Hoffmann TJ, Passarelli MN, Graff RE, Emami NC, Sakoda LC, Jorgenson E, Habel LA, Shan J, Ranatunga DK, Quesenberry CP *et al.* (2017) Genome-wide association study of prostate-specific antigen levels identifies novel loci independent of prostate cancer. Nat Commun 8:14248.

Horvat M, Potocnik U, Repnik K, Kavalar R, Zadnik V, Potrc S and Stabuc B (2017) Single nucleotide polymorphisms in genes MACC1, RAD18, MMP7 and SDF-1a as prognostic factors in resectable colorectal cancer. Radiol Oncol 51:151-159.

Hou Q, Li MY, Huang WT, Wei FF, Peng JP, Lou MW and Qiu JG (2017) Association between three VEGF polymorphisms and renal cell carcinoma susceptibility: A meta-analysis. Oncotarget 8:50061-50070.

Jeon YJ, Kim JW, Park HM, Jang HG, Kim JO, Oh J, Chong SY, Kwon SW, Kim EJ, Oh D *et al.* (2014) Interplay between 3′-UTR polymorphisms in the vascular endothelial growth factor (VEGF) gene and metabolic syndrome in determining the risk of colorectal cancer in Koreans. BMC Cancer 14:881.

Jia YM, Xie YT, Wang YJ, Han JY, Tian XX and Fang WG (2015) Association of genetic polymorphisms in CDH1 and CTNNB1 with breast cancer susceptibility and patients' prognosis among Chinese Han women. PLoS One 10:e0135865.

Jin EH, Kim J, Lee S and Hong JH (2015) Association between polymorphisms in APE1 and XRCC1 and the risk of gastric cancer in Korean population. Int J Clin Exp Med 8:11484-11489.

Jones CC, Bradford Y, Amos CI, Blot WJ, Chanock SJ, Harris CC, Schwartz AG, Spitz MR, Wiencke JK, Wrensch MR *et al.* (2019) Cross-cancer pleiotropic associations with lung cancer risk in African Americans. Cancer Epidemiol Biomarkers Prev 28:715-723.

Kamiza AB, Kamiza S, Singini MG and Mathew CG (2020) Association of TP53 rs1042522 with cervical cancer in the sub-Saharan African population: A meta-analysis. Trop Med Int Health 25:666-672.

Kesh K, Subramanian L, Ghosh N, Gupta V, Gupta A, Bhattacharya S, Mahapatra NR and Swarnakar S (2015) Association of MMP7−181A→G promoter polymorphism with gastric cancer risk: Influence of nicotine in differential allele-specific transcription via increased phosphorylation of cAMP-response element-binding protein (Creb). J Biol Chem 290:14391-14406.

Kristiansen W, Karlsson R, Rounge TB, Whitington T, Andreassen BK, Magnusson PK, Fosså SD, Adami HO, Turnbull C, Haugen TB *et al.* (2015) Two new loci and gene sets related to sex determination and cancer progression are associated with susceptibility to testicular germ cell tumor. Hum Mol Genet 24:4138-4146.

Li Q, Ma R and Zhang M (2018) XRCC1 rs1799782 (C194T) polymorphism correlated with tumor metastasis and molecular subtypes in breast cancer. Onco Targets Ther 11:8435-8444.

Li W, Zhang M, Huang C, Meng J, Yin X and Sun G (2019) Genetic variants of DNA repair pathway genes on lung cancer risk. Pathol Res Pract 215:152548.

Li YK, Xu Q, Sun LP, Gong YH, Jing JJ, Xing CZ and Yuan Y (2020) Nucleotide excision repair pathway gene polymorphisms are associated with risk and prognosis of colorectal cancer. World J Gastroenterol 26:307-323.

Linhares P, Viana-Pereira M, Ferreira M, Amorim J, Nabiço R, Pinto F, Costa S, Vaz R and Reis RM (2018) Genetic variants of vascular endothelial growth factor predict risk and survival of gliomas. Tumour Biol 40:1010428318766273.

Liu D, Zheng Y, Wang M, Deng Y, Lin S, Zhou L, Yang P, Dai C, Xu P, Hao Q, Song D *et al.* (2018) Four common polymorphisms of BRIP1 (rs2048718, rs4988344, rs4986764, and rs6504074) and cancer risk: evidence from 13,716 cancer patients and 15,590 cancer-free controls. Aging (Albany NY), 10:266-277.

Liu GC, Zhou YF, Su XC and Zhang J (2019) Interaction between TP53 and XRCC1 increases susceptibility to cervical cancer development: A case control study. BMC Cancer 19:24.

Liu P, Zhuo ZJ, Zhu J, Yang Z, Xin Y, Li S, Li L, Li Y, Wang H and He J (2020) Association of TP53 rs1042522 C>G and miR-34b/c rs4938723 T>C polymorphisms with hepatoblastoma susceptibility: A seven-center case–control study. J Gene Med 22:e3182.

Liu R, Ning L, Liu X, Zhang H, Yu Y, Zhang S, Rao W, Shi J, Sun H, and Yu Q (2017) Association between single nucleotide variants of vascular endothelial growth factor A and the risk of thyroid carcinoma and nodular goiter in a Han Chinese population. Oncotarget 8:15838-15845.

Ma H, Yu H, Liu Z, Wang LE, Sturgis EM and Wei Q (2012) Polymorphisms of XPG/ERCC5 and risk of squamous cell carcinoma of the head and neck. Pharmacogenet Genomics 22:50-57.

Ma XD, Cai GQ, Zou W, Huang YH, Zhang JR, Wang DT and Chen BL (2013) First evidence for the contribution of the genetic variations of BRCA1-interacting protein 1 (BRIP1) to the genetic susceptibility of cervical cancer. Gene 524:208-213.

Mechanic LE, Bowman ED, Welsh JA, Khan MA, Hagiwara N, Enewold L, Shields PG, Burdette L, Chanock S and Harris CC (2007) Common genetic variation in TP53 is associated with lung cancer risk and prognosis in African Americans and somatic mutations in lung tumors. Cancer Epidemiol Biomarkers Prev 16:214-222.

Minina VI, Bakanova ML, Soboleva OA, Ryzhkova AV, Titov RA, Savchenko YA, Sinitsky MY, Voronina EN, Titov VA and Glushkov AN (2019) Polymorphisms in DNA repair genes in lung cancer patients living in a coal-mining region. Eur J Cancer Prev 28:522-528.

Nikolić ZZ, Branković AS, Savić-Pavićević DL, Preković SM, Vukotić VD, Cerović SJ, Filipović NN, Tomović SM, Romac SP and Brajušković GN (2014) Assessment of association between common variants at 17q12 and prostate cancer risk-evidence from Serbian population and meta-analysis. Clin Transl Sci 7:307-313.

Oh JJ, Kim TJ, Lee IJ, Song BD, Lee DH, Jung YS, Lee HM, Hong SK, Lee S, Ho JN *et al.* (2017) MP33-19 An Exome-wide association study replicated for prostate cancer in Korean population. J Urology 197:e424-e425.

Oussalah A, Avogbe PH, Guyot E, Chery C, Guéant-Rodriguez RM, Ganne-Carrié N, Cobat A, Moradpour D, Nalpas B, Negro F *et al.* (2017) BRIP1 coding variants are associated with a high risk of hepatocellular carcinoma occurrence in patients with HCV-or HBV-related liver disease. Oncotarget 8:62842-62857.

Ozola A, Ruklisa D and Pjanova D (2019) The complementary effect of rs1042522 in TP53 and rs1805007 in MC1R is associated with an elevated risk of cutaneous melanoma in Latvian population. Oncol Lett 18:5225-5234.

Painter JN, O'Mara TA, Batra J, Cheng T, Lose FA, Dennis J, Michailidou K, Tyrer JP, Ahmed S, Ferguson K *et al.* (2015) Fine-mapping of the HNF1B multicancer locus identifies candidate variants that mediate endometrial cancer risk. Hum Mol Genet 24:1478-1492.

Pouladi N, Abdolahi S, Farajzadeh D and Hosseinpour Feizi MA (2019) Haplotype and linkage disequilibrium of TP53-WRAP53 locus in Iranian-Azeri women with breast cancer. PLoS One 14:e0220727.

Qiao L, Feng X, Wang G, Zhou B, Yang Y and Li M (2018) Polymorphisms in BER genes and risk of breast cancer: Evidences from 69 studies with 33760 cases and 33252 controls. Oncotarget 9:16220-16233.

Ren LP, Xian YS, Diao DM, Chen Y, Guo Q and Dang CX (2013) Further evidence for the contribution of the BRCA1-interacting protein-terminal helicase 1 (BRIP1) gene in breast cancer susceptibility. Genet Mol Res 12:5793-5801.

Ricci-Vitiani L, Pallini R, Biffoni M, Todaro M, Invernici G, Cenci T, Maira G, Parati EA, Stassi G, Larocca LM *et al.* (2010) Tumour vascularization via endothelial differentiation of glioblastoma stem-like cells. Nature 468:824-828.

Ricks-Santi L, McDonald JT, Gold B, Dean M, Thompson N, Abbas M, Wilson B, Kanaan Y, Naab TJ and Dunston G (2017) Next Generation sequencing reveals high prevalence of BRCA1 and BRCA2 variants of unknown significance in early-onset breast cancer in African American Women. Ethn Dis 27:169-178.

Ríos-Tamayo R, Lupiañez CB, Campa D, Hielscher T, Weinhold N, Martínez-López J, Jerez A, Landi S, Jamroziak K, Dumontet C *et al.* (2016) A common variant within the HNF1B gene is associated with overall survival of multiple myeloma patients: Results from the IMMEnSE consortium and meta-analysis. Oncotarget 7:59029-59048.

Roberts MR, Shields PG, Ambrosone CB, Nie J, Marian C, Krishnan SS, Goerlitz DS, Modali R, Seddon M, Lehman T *et al.* (2011) Single-nucleotide polymorphisms in DNA repair genes and association with breast cancer risk in the web study. Carcinogenesis 32:1223-1230.

Ru JY, Cong Y, Kang WB, Yu L, Guo T and Zhao JN (2015) Polymorphisms in TP53 are associated with risk and survival of osteosarcoma in a Chinese population. Int J Clin Exp Pathol 8:3198-3203.

Sagna T, Bonora E, Ouedraogo MNL, Fusco D, Zoure AA, Bisseye C, Djigma F, Kafando JG, Zongo N, Douamba Z *et al.* (2019) Identification of BRCA1/2 p. Ser1613Gly, p. Pro871Leu, p. Lys1183Arg, p. Glu1038Gly, p. Ser1140Gly, p. Ala2466Val, p. His2440Arg variants in women under 45 years old with breast nodules suspected of having breast cancer in Burkina Faso. Biomol Concepts 10:120-127.

Salimzadeh H, Lindskog EB, Gustavsson B, Wettergren Y and Ljungman D (2020) Association of DNA repair gene variants with colorectal cancer: Risk, toxicity, and survival. BMC Cancer 20:409.

Setiawan VW, Haessler J, Schumacher F, Cote ML, Deelman E, Fesinmeyer MD, Henderson BE, Jackson RD, Vöckler JS, Wilkens LR *et al.* (2012) HNF1B and endometrial cancer risk: Results from the PAGE study. PLoS One 7:e30390.

Sharma KL, Misra S, Kumar A and Mittal B (2012) Higher risk of matrix metalloproteinase (MMP-2, 7, 9) and tissue inhibitor of metalloproteinase (TIMP-2) genetic variants to gallbladder cancer. Liver Int 32:1278-1286.

Shi J, Tong J, Cai S, Qu X and Liu Y (2013) Correlation of the BACH1 Pro919Ser polymorphism with breast cancer risk: A literature-based meta-analysis and meta-regression analysis. Exp Ther Med 6:435-444.

Smolarz B and Romanowicz H (2018) Association between single nucleotide polymorphism of DNA repair genes and endometrial cancer: A case-control study. Int J Clinical Exp Pathol 11:1732-1738.

Smolarz B, Michalska MM, Samulak D, Romanowicz H and Wójcik L (2019) Polymorphism of DNA repair genes in breast cancer. Oncotarget 10:527-535.

Song X, Wang S, Hong X, Li X, Zhao X, Huai C, Chen H, Gao Z, Qian J, Wang J *et al.* (2017) Single nucleotide polymorphisms of nucleotide excision repair pathway are significantly associated with outcomes of platinum-based chemotherapy in lung cancer. Sci Rep 7:11785.

Song Y, Yang Y, Liu L and Liu X (2019) Association between five polymorphisms in vascular endothelial growth factor gene and urinary bladder cancer risk: A systematic review and meta-analysis involving 6671 subjects. Gene 698:186-197.

Tavares CB, Alves-Ribeiro FA, Nery Junior EDJ, de Vasconcelos-Valença RJ, Campos-Verdes LC, Gomes FDCSA, Lopes-Costa PV, Santos AR, Pinho-Sobral AL, Campelo V *et al.* (2020) Association of XRCC1 rs1799782 and ERCC2 rs13181 polymorphisms with glioma risk: A systematic review and meta-analysis. Research Square. DOI: 10.21203/rs.3.rs-56338/v1.

Tong Y, Qu Y, Li S, Zhao F, Wang Y and Mu D (2018) Cumulative evidence for relationships between multiple variants of HNF1B and the risk of prostate and endometrial cancers. BMC Med Genet 19:128.

Wang S, Qian F, Zheng Y, Ogundiran T, Ojengbede O, Zheng W, Blot W, Nathanson KL, Hennis A, Nemesure B *et al.* (2018) Genetic variants demonstrating flip-flop phenomenon and breast cancer risk prediction among women of African ancestry. Breast Cancer Res Treat 168:703–12.

Wieczorek E, Wasowicz W, Gromadzinska J and Reszka E (2014) Functional polymorphisms in the matrix metalloproteinase genes and their association with bladder cancer risk and recurrence: A mini-review. Int J Urol 21:744-752.

Wu H, Qiao N, Wang Y, Jiang M, Wang S, Wang C and Hu L (2013) Association between the telomerase reverse transcriptase (TERT) rs2736098 polymorphism and cancer risk: Evidence from a case-control study of non-small-cell lung cancer and a meta-analysis. PLoS One 8:e76372.

Xie B, Zhang Z, Wang H, Chen Z, Wang Y, Liang H, Yang G, Yang X and Zhang H (2016) Genetic polymorphisms in MMP 2, 3, 7, and 9 genes and the susceptibility and clinical outcome of cervical cancer in a Chinese Han population. Tumour Biol 37:4883-4888.

Yang F, Mu X, Bian C, Zhang H, Yi T, Zhao X and Lin X (2019) Association of excision repair cross-complimentary group 1 gene polymorphisms with breast and ovarian cancer susceptibility. J Cell Biochem 120:15635-15647.

Yang P, Kollmeyer TM, Buckner K, Bamlet W, Ballman KV and Jenkins RB (2005) Polymorphisms in GLTSCR1 and ERCC2 are associated with the development of oligodendrogliomas. Cancer 103:2363-2372.

Yin J, Vogel U, Wang H, Ma Y, Wang C, Liang D, Liu J, Yue L, Zhao Y and Ma J (2013) HapMap-based study identifies risk sub-region on chromosome 19q13. 3 in relation to lung cancer among Chinese. Cancer Epidemiol 37:923-929.

Zhang G, Xu Q, Wang Z, Sun L, Lv Z, Liu J, Xing C and Yuan Y (2019) p53 protein expression affected by TP53 polymorphism is associated with the biological behavior and prognosis of low rectal cancer. Oncol Lett 18:6807-6821.

Zhao Y, Zhao E, Zhang J, Chen Y, Ma J and Li H (2019) A comprehensive evaluation of the association between polymorphisms in XRCC1, ERCC2, and XRCC3 and prognosis in hepatocellular carcinoma: A meta-analysis. J Oncol 2019:2408946.

Zhu G, Su H, Lu L, Guo H, Chen Z, Sun Z, Song R, Wang X, Li H and Wang Z (2016) Association of nineteen polymorphisms from seven DNA repair genes and the risk for bladder cancer in Gansu province of China. Oncotarget 7:31372-31383.

Zhu J, Qi P and Li Z (2018) Interaction Between XRCC1 Gene polymorphisms and obesity on susceptibility to papillary thyroid cancer in Chinese Han population. Cell Physiol Biochem 49:638-644.

Zhu LX, Ye XJ, Wang YG, Zhu JJ, Xie WZ, Zhao YM and Lai XY (2015) 3'-UTR polymorphism (rs10434) in the VEGF gene is associated with B-CLL in a Chinese population. Genet Mol Res 14:4085-4089.
